# CSF metabolomic signature during therapy for childhood acute lymphoblastic leukemia predicts subsequent working memory impairment

**DOI:** 10.1186/s10020-025-01414-z

**Published:** 2025-12-30

**Authors:** Jeremy Willekens, Sameera Ramjan, Stephen A. Sands, Yongkyu Park, Nirajan K.C., Melissa A. Burns, Jennifer J. G. Welch, Justine Kahn, Kara M. Kelly, Thai-Hoa Tran, Bruno Michon, Lisa Gennarini, Andrew Place, Lewis B. Silverman, Peter D. Cole

**Affiliations:** 1 Rutgers Cancer Institute, Division of Pediatric Hematology/Oncology, New Brunswick, NJ USA; 2 Department of Psychiatry & Behavioral Sciences, Memorial Sloan Kettering Cancer Center, New York, NY USA; 3 Department of Pediatrics, Memorial Sloan Kettering Cancer Center, New York, NY USA; 4 Dana-Farber/Boston Children’s Cancer and Blood Disorders Center; Harvard Medical School, Boston, MA USA; 5 Division of Pediatric Hematology/Oncology, Alpert School of Medicine, Brown University, Providence, RI USA; 6 Division of Pediatric Hematology, Oncology and Stem Cell Transplantation, Columbia University Medical Center, New York, NY USA; 7 Department of Pediatrics, Roswell Park Comprehensive Cancer Center, University at Buffalo Jacobs School of Medicine and Biomedical Sciences, Buffalo, NY USA; 8 Division of Pediatric Hematology Oncology, Charles-Bruneau Cancer Center, CHU Sainte-Justine, Université de Montréal, Montréal, Québec Canada; 9 Centre Hospitalier Universitaire de Quebec, Quebec City, QC Canada; 10 Children’s Hospital at Montefiore, Bronx, NY USA; 11 Department of Pediatrics, Rutgers Robert Wood Johnson Medical School, New Brunswick, NJ USA

**Keywords:** Pediatric Acute Lymphoblastic Leukemia, Chemotherapy-Related Cognitive Impairment, Chemobrain, Metabolomics, Biomarker, Neurocognitive functioning, Survivorship, Glycerophospholipid metabolism, Cerebrospinal fluid

## Abstract

**Background:**

Although typically curative, treatment for pediatric acute lymphoblastic leukemia (ALL) is associated with neurotoxicity and leads to chemotherapy-related cognitive impairment (CRCI) in 40–70% of survivors. Cerebrospinal fluid (CSF), which is routinely collected during intrathecal chemotherapy, offers a direct window into brain metabolism. This study characterizes longitudinal metabolic changes in the CSF of pediatric patients undergoing chemotherapy for ALL.

**Methods:**

CSF samples from 45 pediatric patients enrolled on the multi-institutional Dana-Farber Cancer Institute (DFCI) ALL Consortium Protocol 16–001 were collected at five standardized timepoints over the first 20 weeks of treatment and analyzed using untargeted metabolomics. Cognitive outcomes were assessed post-treatment using age-appropriate Wechsler Intelligence scales, with the Working Memory Index (WMI) serving as the primary cognitive measure. Patients with WMI scores at least one standard deviation above (*n* = 21) or below (*n* = 24) the mean were selected for metabolomic comparison. This study constitutes an exploratory aim of the 16–001 clinical trial.

**Results:**

Our analysis revealed a profound reorganization of the CSF metabolome during the first 18 days of treatment, spanning the induction phase of chemotherapy and early leukemia remission. This shift was characterized by alterations in amino acid, phospholipid, and one-carbon metabolism. Moreover, we identified a lipid-rich metabolomic signature predictive of low post-treatment WMI, implicating metabolic dysregulation in CRCI susceptibility.

**Conclusions:**

These findings highlight the dynamic impact of chemotherapy on the CSF metabolome and support its utility as a matrix for monitoring neurotoxicity during pediatric ALL therapy. CSF metabolomics may enable the early identification of patients at risk for CRCI through predictive biomarkers and guide future neuroprotective interventions.

Trial registration: Dana-Farber Cancer Institute ALL Consortium Protocol 16–001, clinicaltrials.gov ID NCT03020030; study start date 03/03/2017.

**Supplementary Information:**

The online version contains supplementary material available at 10.1186/s10020-025-01414-z.

## Background

Over the last several decades, cure rates for childhood ALL have improved and are now approximately 90% (Hunger and Mullighan 2015); however, the late effects of therapy remain a concern. Even with the omission of cranial radiation for nearly all patients in contemporary regimens, neurocognitive impairment is frequently observed in long-term survivors. Given that the peak age of incidence for childhood ALL is between the ages of 2 and 5 years, the developing brains of these young patients are particularly susceptible to neurotoxic damage from chemotherapy. Consequently, 40–70% of childhood ALL survivors exhibit cognitive deficits in multiple domains, including attention, processing speed, executive function, and working memory (Iyer et al. 2015; Egset et al. 2024; Plas et al. 2021; Mavrea et al. 2021). Notably, CRCI presents with a wide spectrum of interpatient variability, with some survivors showing intact cognitive function, whereas others appear to be more susceptible to cognitive decline (Williams et al. 2016). The early identification of this subset of patients who are more susceptible to CRCI is of paramount importance in preserving the long-term quality of life of childhood ALL survivors. Alternative approaches to prevent or mitigate CRCI remain limited, despite increasing awareness of this significant problem (Nguyen and Ehrlich 2020). Therefore, prioritizing early detection and advancing our understanding of CRCI pathophysiology is crucial.

During ALL treatment, chemotherapy is administered intrathecally at regular intervals to bypass the blood–brain barrier and prevent leukemia relapses within the central nervous system. Cerebrospinal fluid (CSF), which is routinely collected during treatment in volumes matching each intrathecal dose, closely surrounds the brain, has highly dynamic turnover, and plays a key role in maintaining central nervous system (CNS) homeostasis. CSF provides a direct window into brain metabolism and is a widely used matrix for biomarker discovery in neurological and neurodegenerative diseases (Krsmanovic et al. 2022; Park et al. 2024 Jun 7; Park, et al. 2025). Consequently, investigating metabolic changes in CSF composition over time may provide key insights into the pathophysiology of CRCI. Advances in omics technologies have established CSF metabolomic profiling as a powerful approach to characterize treatment-related alterations, identify candidate biomarkers, and predict cognitive outcomes. Because chemotherapy is delivered in distinct treatment phases, serial CSF sampling can capture metabolic changes at key timepoints throughout treatment.

Recent improvements in metabolome databases have enabled not only the association of individual metabolites with deleterious phenotypes but also their integration into broader metabolic networks. To analyze such complex datasets, various statistical and machine learning (ML) models have been developed. While classical statistical models are designed to identify significant differences between conditions, ML models are optimized to predict phenotypes by learning complex patterns within the data. When implemented in a longitudinal design across treatment phases, CSF metabolomic profiling allows dynamic biochemical trajectories to be resolved over time. Hence, longitudinal CSF metabolomics offers a systems-level framework for exploring molecular signatures potentially linked to treatment-induced cognitive decline.

Early alterations in CSF composition may reflect treatment-induced neurotoxicity that precedes and contributes to the emergence of cognitive symptoms. However, CRCI impacts multiple cognitive domains to varying degrees, resulting in considerable interpatient variability among childhood ALL survivors. Therefore, targeting individual cognitive domains is a promising strategy for delineating the specific effects of chemotherapy on cognitive function. For example, working memory deficits, which are linked to learning disorders, have been documented in children treated for ALL without irradiation (Kanellopoulos et al. 2016). Additionally, working memory defects are among the most prominent late effects of both leukemia and chemotherapy and have a significant downstream impact on survivorship and education (Plas et al. 2021; Maehler and Schuchardt 2016). Given its central role in executive function development and its association with school achievement independent of intelligence, working memory represents a particularly relevant focus for advancing our understanding of CRCI. Our long-term goal is to identify biomarkers associated with treatment-related changes in cognitive function. In this study, we hypothesized that temporal variations in CSF metabolite profiles during ALL treatment are predictive of subsequent working memory deficits.

This study addresses two significant gaps in current research: (1) the scarcity of temporal CSF metabolomic data during ALL treatment in pediatric patients and (2) the absence of reliable predictive biomarkers for CRCI. We conducted metabolomic profiling of CSF from pediatric patients enrolled on a multi-institutional trial (Dana-Farber Cancer Institute (DFCI) ALL Consortium protocol 16–001, NCT03020030). By investigating CSF metabolic changes across five critical timepoints throughout the first 20 weeks of chemotherapy, we aimed to uncover metabolic alterations linked to cognitive deficits. We characterized longitudinal CSF metabolic alterations using a combination of pathway topology and enrichment analyses. Given that working memory has been extensively shown to be negatively impacted by treatment for ALL (Kanellopoulos et al. 2016; Reddick and Conklin 2010; Insel et al. 2017; Firoozi and Azadfar 2017; Plas et al. 2015), the working memory index (WMI) subtests were administered 1–2 years after therapy to evaluate the cognitive outcomes associated with these metabolic alterations. Using an interpretable logistic regression–based ML model, we identified a metabolomic signature predictive of low WMI in this patient cohort based on CSF metabolite abundances measured at 12 weeks post-diagnosis, when interventions may still be effective in preventing or mitigating treatment-related toxicity. These findings offer a novel, metabolomics-based approach for the early detection of CRCI and may guide the development of targeted interventions to mitigate cognitive decline in childhood cancer survivors.

## Methods

### Patient enrollment and biospecimen collection

**Fig. 1:**
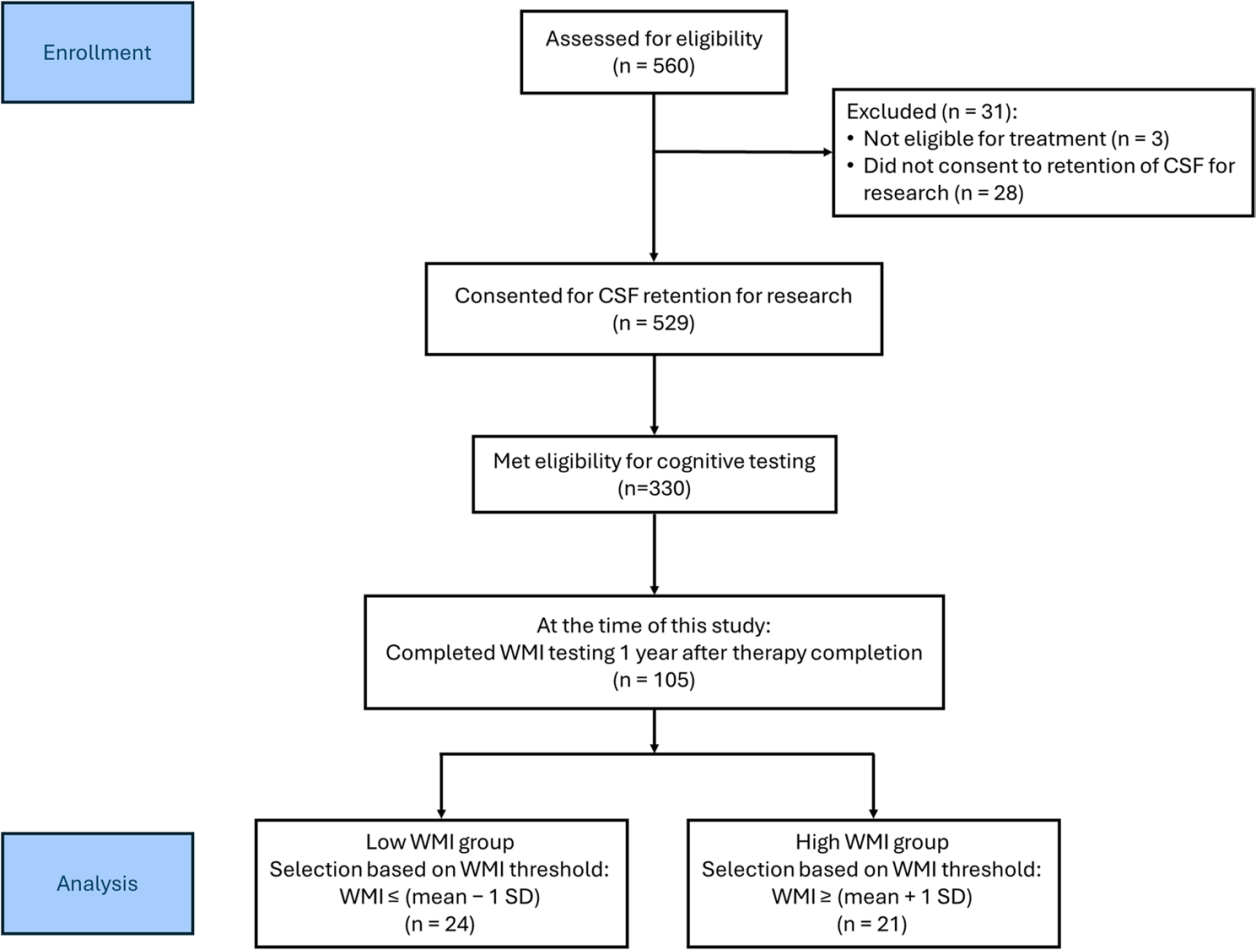
Flow diagram of patient enrollment and WMI-based group selection. Flowchart illustrating patient enrollment, cognitive assessment one year off therapy, and selection of patients into low and high Working Memory Index (WMI) groups based on predefined thresholds. CSF: Cerebrospinal fluid; SD: standard deviation

CSF samples were collected from patients enrolled in the DFCI ALL Consortium Protocol 16–001, clinicaltrials.gov ID NCT03020030. A total of 560 patients aged 1–21 years with newly diagnosed ALL were enrolled in this study between March 3, 2017, and November 10, 2022, across eight sites in North America (Fig. 1). The participating sites were Dana-Farber/Boston Children’s Hospital (Boston, MA); Columbia University Medical Center (New York, NY); Hasbro Children’s Hospital (Providence, RI); Montefiore Medical Center (Bronx, NY); Roswell Park Cancer Institute (Buffalo, NY); Rutgers Cancer Institute of New Jersey (New Brunswick, NJ); CHU de Québec—Université Laval (Québec City, QC, Canada); and CHU Sainte-Justine (Montreal, QC, Canada). Institutional review board approval was obtained at each site for the treatment protocol (Supp. Table 1) and ancillary studies. Informed consent was provided by the participants’ guardians or by patients over the age of 18. Written assent was also obtained from the pediatric patients on the basis of institutional guidelines. All procedures were conducted in accordance with relevant ethical regulations and the approvals of the participating institutional review boards.

### Neuropsychological measurement

All patients provided informed consent and assent, following local institutional review board guidelines, for neurocognitive testing to be performed one year after the completion of therapy. The neuropsychological assessment included a two-hour test battery for patients and a parent-reported questionnaire evaluating executive function. Evaluations were conducted in an outpatient setting 9–24 months post-treatment. Specifically, age-appropriate intellectual testing was administered: the Wechsler Preschool & Primary Scale of Intelligence – Fourth Edition (WPPSI-IV) for ages 2–5 (Wechsler 2012), the Wechsler Intelligence Scale for Children – Fifth Edition (WISC-V) for ages 6–15 (Wechsler 2014), and the Wechsler Adult Intelligence Scale – Fourth Edition (WAIS-IV) for patients over age 16 (Wechsler 2008). The WMI serves as the primary measure for evaluating the association between cognitive function and chemotherapy. On the basis of the availability of CSF samples across all timepoints, 24 patients with a WMI at least one standard deviation below the mean (100) 1–2 years after chemotherapy were compared with 21 patients with a WMI at least one standard deviation above the mean (Fig. 1).

### Sample storage, preparation, and data extraction

No formal statistical analysis plan for this exploratory study was pre-specified within the clinical trial study protocol at the time of protocol development. Thus, CSF samples were obtained only for patients who consented to the optional retention of leftover CSF for research and the patient selection was based on the WMI (Fig. 1). Metabolite abundance was measured in CSF collected prior to the administration of intrathecal chemotherapy at the five timepoints (T0–T4) indicated in Table 1 and Supp. Table 1. Consent to collect leftover CSF prior to chemotherapy (T0) was also obtained from patients diagnosed with ALL before the first therapeutic lumbar puncture. As per standard of care, CSF was removed in a volume approximately equal to the volume of chemotherapy to be administered (typically 5–7 mL). Between 0.5 and 1 mL was sent for routine analyses of cell count and cytospin. For consenting patients, the remaining CSF was placed on ice immediately and centrifuged within one hour to remove cellular elements. The supernatant was frozen at −80 °C and shipped on dry ice to the Rutgers Cancer Institute. Upon receipt and verification, 100 µL aliquots were sent to Metabolon, Inc., on dry ice. Global, untargeted metabolite profiles were generated via ultrahigh-performance liquid chromatography-tandem mass spectrometry (UPLC-MS/MS). Standardized in-house quality control protocols were utilized by Metabolon to account for instrument and sample variability (Ford et al. 2020, 2022).

**Table 1:** **CSF** sampling schedule and working memory assessment at the end of therapy

Treatment phase	Approximate time since start of therapy	CSF Collection
Pretreatment	**0 days**	**T0**
Induction 1A	**18 days**	**T1**
	32 days	
Consolidation 1A	10 weeks	
CNS phase	**11 weeks**	**T2**
	**12 weeks**	**T3**
	14 weeks	
Consolidation 2	**20 weeks**	**T4**
Continuation	41 weeks	
End of therapy	25 months	
1–2 years off therapy	**34–49 months**	**WMI assessment**

*CSF* Cerebrospinal fluid, *WMI* Working Memory Index

### Compound identification and curation

The raw data extraction, peak identification and quality control processes were performed via proprietary hardware and software (Metabolon Inc., Durham, NC, USA). Metabolites were identified using an in-house proprietary library constructed from purified standards. Each standard entry includes the retention time/index (RI), mass-to-charge ratio (m/z), and chromatographic data, including MS/MS spectral information. Biochemical identifications are based on three criteria: retention index within a narrow RI window of the proposed identification, accurate mass match to the library ± 10 ppm, and MS/MS forward and reverse match scores with authentic standards. Curation procedures were also performed internally by Metabolon via proprietary visualization and interpretation software. This included verification of peak identification consistency across samples and library matches. Peaks were quantified using area under the curve. Volume-normalized metabolite abundance data were used for subsequent analyses.

### Statistical and bioinformatics analysis

#### Data preprocessing and visualization

Individual metabolite abundance was log10-transformed and standardized to zero mean and unit variance using the scikit-learn Python package (Pedregosa et al. 2011), version 1.6.1. Exploratory data analysis (EDA) was conducted to visualize the distribution of metabolite abundance across groups. Kernel density estimation plots and box plots were generated using the seaborn Python package (Waskom 2021), version 0.13.2, to visualize metabolite distribution patterns across groups.

#### Principal component analysis (PCA)

Principal component analysis (PCA) was performed for dimensionality reduction and visualization of global variation in CSF metabolite profiles. PCA was applied to the log10-transformed, standardized metabolite matrix using the scikit-learn Python package (Pedregosa et al. 2011), version 1.6.1. The first two principal components (PC1 and PC2) were retained for visualization and plotted as two-dimensional score plots. Samples were colored according to the variable of interest (timepoint or WMI status). Group-level dispersion was visualized using Hotelling’s T^2^ confidence ellipses. The percentage of variance explained by each principal component was reported on the corresponding axis labels.

#### Uniform manifold approximation and projection (UMAP) for dimension reduction

To assess sample similarity in the high-dimensional space, UMAP dimensionality reduction and visualization were performed on the standardized dataset using the umap (McInnes et al. 2018), version 0.1.1, Python package. First, principal component analysis (PCA, 50 components) was applied as a preprocessing step, as recommended by the UMAP Python package documentation, followed by UMAP dimensionality reduction. Default hyperparameters were used. The resulting embeddings were visualized in two-dimensional space, with data points colored by group label (timepoint, WMI, or CNS status) to evaluate whether the separation of samples reflected their biological grouping.

#### Pathway analysis 

Pathway topological analysis was performed using the MetaboAnalyst 6.0 online platform (http://www.metaboanalyst.ca/) with standard settings (Pang et al. 2024) to identify changes in metabolic pathways over the course of chemotherapy treatment (T0 to T4, Table 1), independent of WMI status. A total of 57 metabolic pathways were analyzed. Pathways with a false discovery rate (FDR) ≤ 0.05 and an impact > 0.10 were retained. Pathway impact plots were generated using the Python package matplotlib (Hunter 2007), version 3.10.0.

#### *Initial feature selection *via* multivariate empirical Bayes analysis (MEBA)*

Initial feature selection (feature filtering) was performed to identify metabolites with differing temporal profiles between the low- and high-WMI groups across four timepoints (T1 to T4). Due to less samples available at T0 (*n* = 16), this timepoint was excluded from this analysis in order to avoid introducing bias in the temporal profiles. The MEBA method was applied via the MetaboAnalyst 6.0 online platform (http://www.metaboanalyst.ca/) (Pang et al. 2024), which computes a T^2^ statistic for each metabolite (Tai and Speed 2006, 2009). This variant of the one-sample Hotelling’s T^2^ was used to rank features on the basis of the strength of evidence for temporal divergence between groups. Importantly, the T^2^ statistic does not provide statistical significance testing and should not be interpreted as a *p* value. Analysis of the top 20 ranked metabolites revealed that the greatest number of metabolites showing significant differences in abundance between groups was observed at T3, 12 weeks post-diagnosis. This observation informed the selection of T3 for the subsequent machine learning analysis.

#### Custom machine learning pipeline for predicting low WMI

Predicting low WMI status was achieved by feeding the log10-transformed standardized data to a custom logistic regression-based ML pipeline (Fig. 3). Briefly, this pipeline relies on stratified repeated K-fold cross validation (n_splits = 4; n_repeats = 5) stratified by WMI status, using the scikit-learn Python package (Pedregosa et al. 2011), version 1.6.1. All available T3 samples were used for model training, with performance estimated through stratified repeated cross-validation rather than a holdout test set. This approach is recommended for limited datasets, as holdout-based validation can produce unstable and biased performance estimates (Tsamardinos 2022; Eertink et al. 2022). The area under the receiver operating characteristic (ROC) curve (AUC) was used as the primary evaluation metric throughout. Recursive feature elimination with cross validation was performed on the top 20 metabolites ranked by the T^2^ statistic at T3 (12 weeks post-diagnosis), and the optimal number of metabolites was determined. The subset of six metabolites yielding the highest AUC was retained after 20 iterations. A hyperparameter tuning step was conducted to identify the optimal penalty type, C value, and, where applicable, l1 ratio (for Elastic Net regularization). Regularization strength (C) was tuned using cross-validation to prevent overfitting and identify the penalty term yielding the best predictive performance. Because logistic regression outputs probabilities, the decision threshold was optimized after model fitting, using the F1 score to balance precision and recall in the context of class imbalance. This workflow follows standard machine-learning practices for small-sample biomarker studies and is not derived from a previously published discovery/validation pipeline. Decision threshold tuning was then performed through 100 iterations by maximizing the F1 score across stratified repeated K-fold cross validation. Beta coefficients were computed both for the initial set of 20 T^2^-ranked metabolites and for the final logistic regression model based on the selected six-metabolite subset. Last, the final model was trained and evaluated using the optimized parameters. The final ROC curve, confusion matrix (true positive rate, false positive rate, false negative rate, and true negative rate), and *p* value were averaged across repeated folds (Willekens 2025). This workflow follows established approaches for predictive modeling, including cross-validation, backward/recursive feature elimination, regularization tuning, and evaluation using ROC and confusion-matrix–based metrics (Dankers et al. 2019). All plots were generated using the matplotlib (Hunter 2007), version 3.10.0, and seaborn (Waskom 2021), version 0.13.2, Python packages.

#### Multivariate inference-based logistic regression

Multivariate logistic regression was applied to the log10-transformed, standardized abundance of the six ML-selected metabolites (1-stearoyl-2-arachidonoyl-GPC (18:0/20:4), 1-arachidonoyl-GPC (20:4n6)*, glycerate, N-acetylasparagine, 1-palmitoyl-2-dihomo-linolenoyl-GPC (16:0/20:3n3 or 6)*, and 1-oleoyl-GPC (18:1)) at T3 (12 weeks post-diagnosis) via the statsmodel (Seabold and Perktold 2010), version 0.14.4, Python package. The model was estimated by maximum likelihood (method = 'MLE') and included no additional covariates to enable comparison of effect estimates with those from the ML-based model. WMI status (low vs. high) was used as the dependent variable. Beta coefficients were calculated, and statistical significance was assessed using the two-sided Wald test. Coefficients were visualized using the matplotlib (Hunter 2007), version 3.10.0, Python package.

## Results

### Participant characteristics and study design

Between 2017 and 2022, a total of 560 patients were enrolled on DFCI 16–001, and 529 provided consent for the optional retention of CSF for research (Fig. 1). Of these, 240 participants (45%) had provided informed consent prior to receiving any intrathecal chemotherapy, allowing collection of the pretreatment (timepoint T0, Table 1) specimen, in addition to the remaining samples collected after the start of protocol therapy (T1–T4). The remaining 289 participants (55%) received their first intrathecal chemotherapy prior to providing informed consent for participation in DFCI 16–001 (Supp. Table 1); CSF was collected from these participants beginning on day 18 of induction (T1) and continued through consolidation 2 (T4). Owing to the lower rate of early consent, the number of CSF samples available at T0 for this metabolomic study was limited (*n* = 16).

This ancillary metabolomic study aimed to identify changes in CSF metabolites associated with low cognitive function at the end of treatment, using a single cognitive metric. Given that working memory is both vulnerable to chemotherapy and commonly targeted by cognitive interventions following ALL treatment, we selected the WMI as a straightforward and clinically relevant measure of potential CRCI (Williams et al. 2016; Stewart et al. 2006; Peterson et al. 2008). Accordingly, samples from 45 patients were selected on the basis of WMI stratification, as measured up to two years after therapy: 21 patients with high WMI (≥ mean + 1 standard deviation, SD) and 24 patients with low WMI (≤ mean −1 SD, Fig. 1). The mean age at diagnosis was not significantly different between the groups (7.9 years for high WMI and 7.2 years for low WMI; Table 2). No group differences were observed in terms of sex or race distribution, although “Hispanic or Latino” patients were overrepresented in the low WMI group (7 of 24) compared with none in the high WMI group (*p* = 0.0102). The immunophenotype, final risk stratification, CNS status, and baseline white blood cell count (WBC) were not significantly associated with WMI group status (Table 2).

**Table 2:** Baseline characteristics of the 45 ALL patients analyzed by untargeted metabolomics

Characteristic	High WMI	Low WMI	*p*-value
	(n = 21)	(n = 24)	
**Age at Diagnosis — year ± SD**	7.9 ± 5.0	7.2 ± 4.3	*p* = 0.8614
**Female Sex — no. (%)**	6 (28.6)	7 (29.2)	*p* > 0.9999
**Race — no. (%)**			*p* = 0.8017
White	18 (85.6)	19 (79.1)	
Black or African American	1 (4.8)	1 (4.2)	
Asian	1 (4.8)	0 (0.0)	
Other	1 (4.8)	3 (12.5)	
More Than One Race	0 (0.0)	1 (4.2)	
**Ethnicity — no. (%)**			*p* = 0.0102
Non-Hispanic	20 (95.2)	16 (66.7)	
Hispanic or Latino	0 (0.0)	7 (29.1)	
Ethnicity Not Known	1 (4.8)	1 (4.2)	
**Immunophenotype — no. (%)**			*p* = 0.1772
B-cell Lineage	18 (85.7)	16 (66.7)	
T-cell Lineage	3 (14.3)	8 (33.3)	
**Final Risk — no. (%)**			*p* = 0.8521
Low Risk	12 (57.1)	12 (50.0)	
Intermediate Risk	5 (23.8)	5 (20.8)	
High Risk	3 (14.3)	6 (25.0)	
Very High Risk	1 (4.8)	1 (4.2)	
**Central Nervous Status (CNS) — no. (%)**			*p* = 0.7050
CNS 1	18 (85.7)	18 (75.0)	
CNS 2	2 (9.5)	5 (20.8)	
CNS 3	1 (4.8)	1 (4.2)	
**White Blood Cell Count Baseline — median [Q1–Q3]**	6.7 [4.2–22.7]	10.9 [5.1–37.5]	*p* = 0.3816

*SD* Standard deviation, *Q1* First quartile, *Q3* Third quartile

### Major shifts in the CSF metabolome occur within the first 18 days post-diagnosis

To better understand the metabolic consequences of chemotherapy over the course of treatment in pediatric ALL patients, CSF samples from all timepoints (Table 1) were subjected to untargeted metabolomic analysis, utilizing UPLC-MS/MS. After curation, the relative abundances of 280 metabolites were determined. Our EDA did not reveal variation in the global metabolomic data distribution based on timepoint (T0–T4), WMI status, or CNS status, demonstrating the absence of major technical or sampling artifacts (Supp. Figure 1). However, visualization of UMAP dimensionality reduction suggested that samples from T1 differed distinctly from those sampled at T0, T2, T3, and T4 (Supp. Figure a). Samples grouping together on the UMAP reflect broad, coordinated shifts occurring across many metabolites rather than changes in only a few. Consequently, the divergence observed at T1 reflects a global reorganization of the CSF metabolome. This finding underscores the importance of the first 18 days of treatment, which corresponds to the chemotherapy induction phase aggressively eliminating malignant cells, as this period leads to significant shifts in the CSF metabolome of pediatric ALL patients. While some changes continue to occur at later timepoints, none reach the magnitude of those observed at between T0 and T1. In contrast, the global data structure does not appear to be influenced by WMI status, as shown by UMAP visualization **(**Supp. Figure b). These findings suggest that although metabolic differences may exist between WMI groups, WMI status does not appear to drive coordinated, global shifts in CSF composition, as treatment timepoint does. We likewise did not observe global separation based on CNS status (Supp. Figure c).

### Amino acid, one-carbon, and lipid metabolism are persistently impacted during treatment

To gain insight into the molecular changes occurring over time in the CSF of pediatric ALL patients undergoing therapy, we performed integrated pathway analysis using the Kyoto Encyclopedia of Genes and Genomes (KEGG) metabolic pathways as the reference knowledge base. We analyzed a total of 57 KEGG pathways and identified 20 selected pathways showing both high statistical significance and substantial topological impact between the first day of treatment (T0) and 20 weeks post-diagnosis (T4). Unlike pathway analysis based on enrichment or over-representation, pathway topology analysis considers the position of each metabolite within the pathway. Thus, if a metabolite occupies a central position in the network, changes in its abundance are more likely to influence the pathway than changes in a terminal end-product. For our analysis, we used the betweenness centrality topology measure, which reflects the relative importance of a metabolite in a given pathway. The results of our pathway analysis are shown in Supp. Figure 5-a, b and summarized in Table 3.

**Table 3:** Summary of metabolic pathways enriched and impacted across all timepoint comparisons

		**T0/T1**		**T1/T2**		**T2/T3**		**T3/T4**	
**Pathway**	**KEGG Pathway ID**	**FDR**	**Impact**	**FDR**	**Impact**	**FDR**	**Impact**	**FDR**	**Impact**
Alanine, aspartate and glutamate metabolism	hsa00250	5.02E-10	0.739	1.78E-19	0.739	1.31E-08	0.739	4.09E-02	0.739
Arginine and proline metabolism	hsa00330	1.41E-05	0.447	1.44E-16	0.447	5.69E-07	0.447	3.76E-03	0.447
Arginine biosynthesis	hsa00220	1.45E-10	0.628	2.39E-17	0.628	9.67E-11	0.628	1.04E-03	0.628
beta-Alanine metabolism	hsa00410	1.18E-12	0.504	7.35E-18	0.504	1.11E-10	0.504	2.63E-06	0.504
Histidine metabolism	hsa00340	1.44E-06	0.221	1.69E-15	0.221	1.71E-08	0.221		
Lysine degradation	hsa00310	4.50E-05	0.125	7.51E-15	0.125	4.53E-02	0.125		
Phenylalanine, tyrosine and tryptophan biosynthesis	hsa00400			1.83E-03	1.000	2.56E-08	1.000	1.03E-07	1.000
Tryptophan metabolism	hsa00380	1.72E-02	0.251	3.06E-04	0.251			2.18E-07	0.251
Glycine, serine and threonine metabolism	hsa00260	6.81E-05	0.600	4.68E-09	0.600	1.51E-03	0.600	2.96E-08	0.600
Taurine and hypotaurine metabolism	hsa00430	2.94E-03	0.429	4.48E-16	0.429	2.09E-04	0.429	1.21E-02	0.429
Cysteine and methionine metabolism	hsa00270			4.62E-05	0.422	5.73E-06	0.422	8.09E-03	0.422
One carbon pool by folate	hsa00670	2.34E-02	0.309	2.44E-06	0.309	4.91E-04	0.309	1.17E-04	0.309
Glycerolipid metabolism	hsa00561	6.43E-05	0.280	2.65E-12	0.280				
Glycerophospholipid metabolism	hsa00564	7.91E-04	0.399	1.23E-20	0.399	7.37E-03	0.399	4.94E-04	0.399
Glutathione metabolism	hsa00480			8.50E-07	0.183	4.71E-03	0.183		
Glyoxylate and dicarboxylate metabolism	hsa00630	2.76E-02	0.190	7.87E-05	0.190	1.49E-02	0.190		
Nicotinate and nicotinamide metabolism	hsa00760	4.09E-13	0.138	2.19E-14	0.138	3.54E-11	0.138		
Phenylalanine metabolism	hsa00360			1.83E-03	0.357	2.56E-08	0.357	1.03E-07	0.357
Pyrimidine metabolism	hsa00240	8.30E-06	0.201	6.06E-08	0.201	2.99E-06	0.201	3.23E-08	0.201
Starch and sucrose metabolism	hsa00500	4.13E-03	0.425	3.17E-09	0.425	3.09E-02	0.425		

This table compiles a selection of metabolic pathways meeting both enrichment (FDR ≤ 0.05) and impact (≥ 0.10) thresholds at all four timepoint comparisons (T0/T1, T1/T2, T2/T3, and T3/T4). For each pathway, the FDR and pathway impact scores are reported for each transition between two timepoints

Interestingly, eight of these 20 pathways were significantly impacted at all timepoints (FDR ≤ 0.05, Table 3). Moreover, ten of the affected pathways are related to various aspects of amino acid metabolism, with six significantly impacted at all timepoints: ‘alanine, aspartate and glutamate metabolism’ (impact = 0.739), ‘arginine and proline metabolism’ (impact = 0.447), ‘arginine biosynthesis’ (impact = 0.628), ‘beta-Alanine metabolism’ (impact = 0.504), ‘glycine, serine and threonine metabolism’ (impact = 0.600), and ‘taurine and hypotaurine metabolism’ (impact = 0.429).

Several of these amino acid pathways intersect with one-carbon metabolism, a critical network of interdependent cycles, including the methionine and folate cycles, which enable transmethylation reactions and nucleotide synthesis. This is the case for ‘glycine, serine and threonine metabolism’ (impact = 0.600), ‘taurine and hypotaurine metabolism’ (impact = 0.429), and ‘cysteine and methionine metabolism’ (impact = 0.422), which are closely linked to the one-carbon metabolism central intermediate homocysteine (Table 3). In addition, our analysis revealed that the ‘one carbon pool by folate’ (impact = 0.309) pathway was also affected. These pathways are impacted at all timepoints, with the exception of ‘cysteine and methionine metabolism’, which is enriched only from T1 to T4. These results are consistent with the fact that methotrexate, an antifolate and key component of modern pediatric ALL chemotherapy regimens, exerts its antineoplastic effects by disrupting one-carbon metabolism, on which the aforementioned pathways critically depend (Peterson et al. 2008; Jannoun 1983; Lofstad et al. 2009; Ficek et al. 2010; Conklin et al. 2012; Cole et al. 2009). Specifically, patients receive intrathecal MTX on days 18 and 32 during Induction IA, and high-dose intravenous MTX during Consolidation 1A (Supp. Table 1).

Finally, our analysis also revealed that ‘glycerolipid metabolism’ was enriched between T0 and T1, as well as between T1 and T2 (impact = 0.280; FDR = 6.43E-05 and 2.65E-12, respectively). Similarly, ‘glycerophospholipid metabolism’ was significantly impacted across all timepoints (impact = 0.399; FDR T0/T1 = 7.91E-04, T1/T2 = 1.23E-20, T2/T3 = 7.37E-03, T3/T4 = 4.94E-04). Although pathway-level enrichment captures broad treatment-associated shifts, examining the distribution of individual metabolites across timepoints can provide a more detailed view of how lipid metabolism is affected. While 18.9% of the total curated metabolites belong to the ‘Lipid’ superpathway, only 10.8% (11 metabolites) show significant changes between T0 and T1, and 13.3% (19 metabolites) between T1 and T2 (Supp. Figure 2-f & Supp. Figure 3-f). However, a notable increase was observed at later stages: 34.8% (24 metabolites) and 38.5% (30 metabolites) of the metabolites showing differential abundance between T2 and T3; and T3 and T4, respectively, were lipids (Supp. Figure 4-f and Supp. Figure 5-f). This initial underrepresentation followed by a marked overrepresentation underscores how dynamically the ‘lipid’ superpathway is affected by chemotherapy treatment over time.

### Patients with a low WMI exhibit consistently lower lipid metabolite levels, especially at T3

**Fig. 2:**
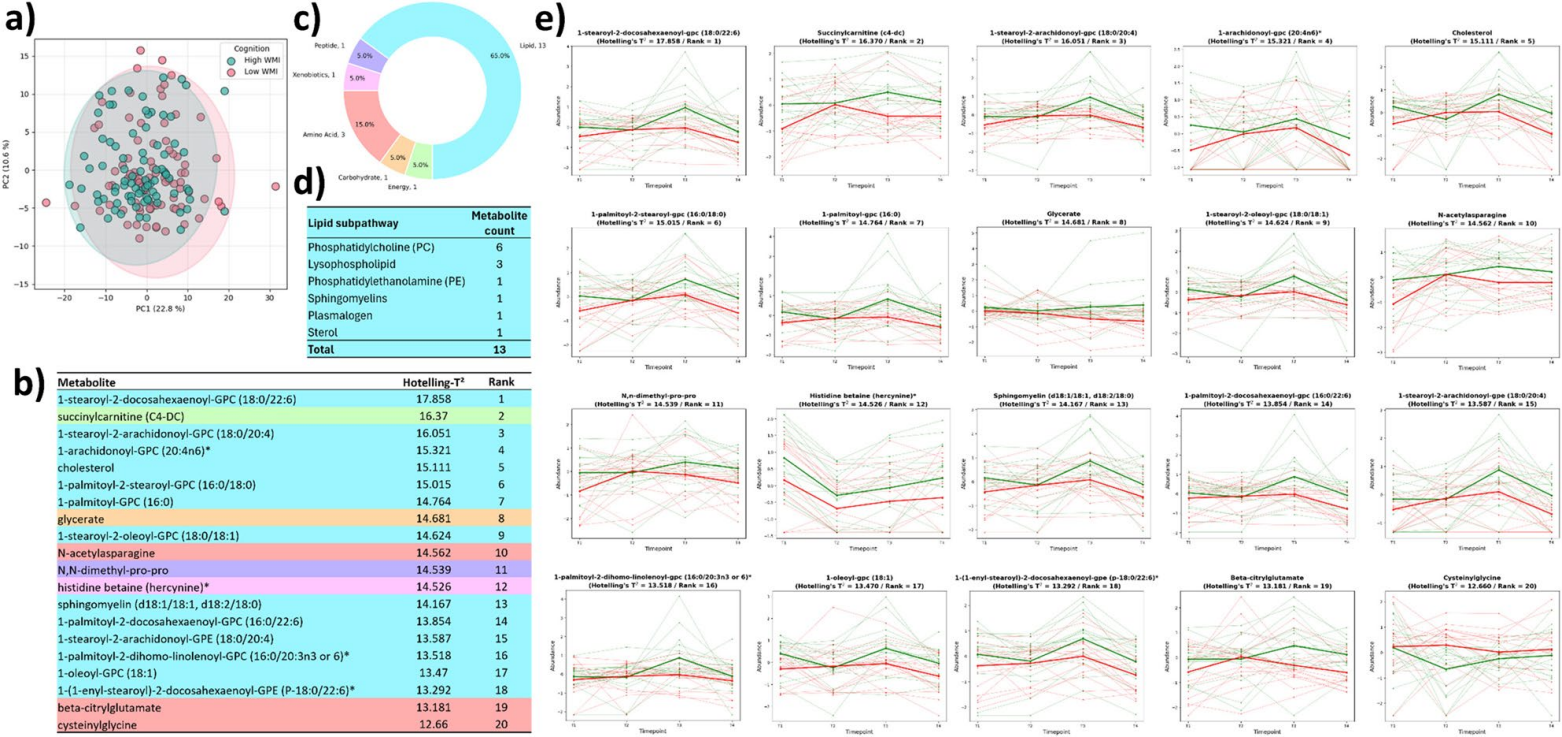
Longitudinal profiles of individual metabolites in the CSF of pediatric leukemia patients stratified by WMI (T1–T4). Principal component analysis (PCA) of all CSF samples across all timepoints, colored by WMI status, is shown in panel **a**). Panel **b**) displays the top 20 metabolites ranked by Hotelling’s T^2^ statistic from multivariate empirical Bayes analysis (MEBA), highlighting those whose temporal abundance patterns differ most between the high- and low-WMI groups across timepoints from T1 (18 days of treatment) to T4 (20 weeks of treatment). Because of the very limited number of T0 samples, baseline data were excluded from this longitudinal assessment. The biochemical classification of these top 20 metabolites is summarized in panel **c**). Thirteen out of 20 belong to the ‘lipid’ superpathway, and their distribution across lipid subpathways is detailed in panel **d**). Temporal trajectories for each of the 20 MEBA-selected metabolites are presented in panel **e**), with low WMI patients in red and high WMI patients in green

Beyond investigating changes in the CSF metabolome of pediatric leukemia patients over time, our overarching goal was to explore how these metabolic changes might be related to cognitive impairment following chemotherapy. The first point of interest lies in the absence of any effect of patients’ CNS status on the global structure of the CSF metabolome, indicating that CNS status does not drive global differences in CSF metabolomic organization, despite the unbalanced distribution of CNS1, CNS2, and CNS3 cases in the cohort (Supp. Figure c). Moreover, while our timepoint-based analysis revealed global shifts in the CSF metabolome, WMI status alone did not discriminate strong global tendencies, as shown by PCA across all samples (Fig. 2-a) and UMAP visualization (Supp. Figure b). Similarly, pathway analysis did not reveal pathway-level differences between patients with a low WMI 1–2 years after treatment completion and those with a high WMI. These approaches aggregate metabolites, which may mask small but biologically relevant changes in single metabolites. Consequently, we shifted our focus to individual metabolite abundance, as their temporal profile may better capture transient metabolic shifts associated with chemotherapy, some of which may underlie cognitive outcomes in pediatric ALL patients.

To do so, we used the MEBA method to rank features based on their longitudinal profile of abundance (Tai and Speed 2006, 2009). MEBA determines a T^2^ statistic as a ranking score to assess whether temporal profiles differ between groups, focusing on ranking features rather than assigning statistical significance via *p* values. Here, we applied MEBA to rank metabolites by their temporal variation between patients with differing WMI at 1–2 years post-treatment to identify candidate metabolic biomarkers associated with cognitive outcomes following chemotherapy. Because T0 samples were limited (n = 16) they were excluded from this analysis to avoid introducing bias in the temporal profiles. MEBA was therefore applied to timepoints T1–T4 only, ensuring that longitudinal differences between WMI groups were evaluated on timepoints with adequate sample representation.

Because of the high dimensionality of omics data, feature filtering is an essential early step of the analysis. Hence, after the metabolites were ranked via Hotelling’s T^2^ statistic from MEBA (Fig. 2-b), we focused on the top 20 metabolites showing the greatest temporal differences between WMI groups. Strikingly, apart from cysteinylglycine, all of these metabolites exhibit a globally lower longitudinal abundance in patients with a low WMI than in those with a high WMI. This difference is particularly pronounced at T3 (Fig. 2-c & Supp. Figure 6) for many of these metabolites (1-stearoyl-2-docosahexaenoyl-GPC (18:0/22:6), succinylcarnitine (C4-DC), cholesterol, 1-palmitoyl-GPC (16:0), 1-stearoyl-2-oleoyl-GPC (18:0/18:1), histidine betaine (hercynine)*, sphingomyelin (d18:1/18:1, d18:2/18:0), 1-palmitoyl-2-docosahexaenoyl-GPC (16:0/22:6), 1-palmitoyl-2-dihomo-linolenoyl-GPC (16:0/20:3n3 or 6)*, 1-oleoyl-GPC (18:1), 1-(1-enyl-stearoyl)-2-docosahexaenoyl-GPE (P-18:0/22:6)*, beta-citrylglutamate). These findings position T3 as the most promising timepoint for identifying biomarkers predictive of low WMI.

Second, among these 20 metabolites, 13 belong to the ‘lipid’ superpathway (Fig. 2-c), suggesting that the metabolic differences between low- and high-WMI patients 1–2 years after therapy extend beyond changes at the level of individual metabolites. Specifically, 6 of these 13 metabolites (Fig. 2-d) are phosphatidylcholines (1-stearoyl-2-docosahexaenoyl-GPC (18:0/22:6), 1-stearoyl-2-arachidonoyl-GPC (18:0/20:4), 1-palmitoyl-2-stearoyl-GPC (16:0/18:0), 1-stearoyl-2-oleoyl-GPC (18:0/18:1), 1-palmitoyl-2-docosahexaenoyl-GPC (16:0/22:6), 1-palmitoyl-2-dihomo-linolenoyl-GPC (16:0/20:3n3 or 6)*) and 3 are lysophospholipids (1-arachidonoyl-GPC (20:4n6)*, 1-palmitoyl-GPC (16:0), 1-oleoyl-GPC (18:1)). While these results provide essential insights into the molecular mechanisms underlying CRCI, a key objective of this study was to develop a model capable of predicting low WMI in our cohort of pediatric ALL patients.

### Multivariate lipid-rich metabolomic signature at T3 predicts low WMI up to two years after therapy completion

**Fig. 3:**
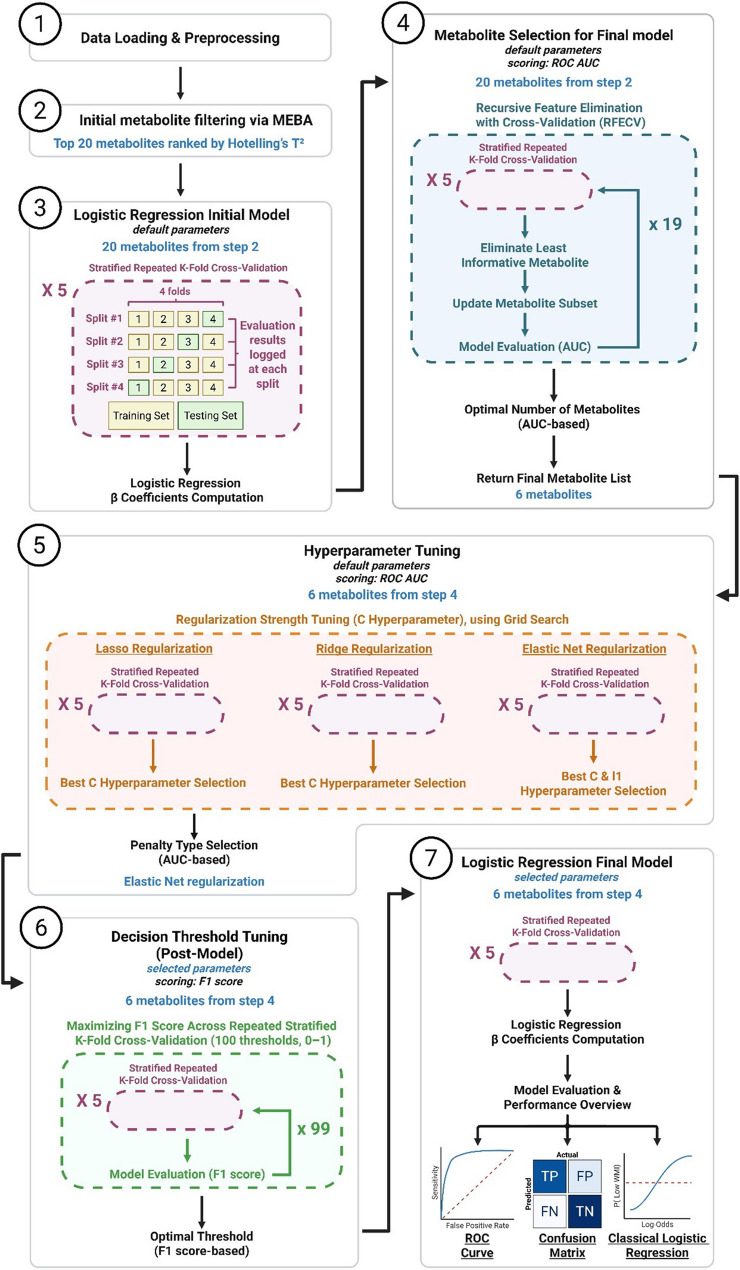
ML pipeline for predicting a low WMI from the CSF metabolome at 12 weeks post-diagnosis. Volume-normalized individual metabolite abundances were (1) log-transformed and standardized by removing the average and scaling to unit variance. The first step of (2) feature filtering uses MEBA to identify a subset of 20 metabolites, which are then used to (3) train and evaluate an initial logistic regression model using stratified repeated k-fold cross-validation. Once the model was fitted, its β coefficients were estimated. To improve the model’s performance, (4) recursive feature elimination with cross-validation was performed, and the optimal number of metabolites was determined using AUC as the scoring metric. The final list of six metabolites yielding the best performance was then returned and used for hyperparameter tuning. (5) The best C hyperparameter (inverse of regularization strength) for each regularization type (L1, LASSO; L2, Ridge; Elastic Net) was determined (with the optimal L1 ratio also tuned for the Elastic Net regularizer), using AUC as the scoring metric. The optimal decision threshold was then (6) determined using the F1 score, which balances precision (the proportion of true positives among predicted positives) and recall (the proportion of true positives among all actual positives). For this step, 100 thresholds were tested (ranging from 0 to 1, with an increment of 0.01). The final logistic regression model was (7) trained and assessed using the selected parameters, and its performance was evaluated by plotting the final ROC curve and confusion matrix. The numbers in parentheses refer to sequential steps in the pipeline diagram. AUC: area under the curve; FN: false negative; FP: false positive; MEBA: multivariate empirical Bayes analysis; ROC: receiver operating characteristic; TN: true negative; TP: true positive

ML is particularly well suited for evaluating the predictive value of biomarkers, as it enables the systematic assessment of how combinations of metabolites can classify individuals into clinically relevant outcome groups. To achieve this goal, we developed an ML model based on the abundance at T3 of the 20 metabolites exhibiting the largest differences between low- and high-WMI patients over time. The model was implemented within a logistic regression framework using WMI-stratified repeated K-fold cross-validation (Fig. 3). Because of the limited number of samples, performance was evaluated through stratified repeated cross-validation rather than relying on a holdout test set, which ensured stable estimation of predictive accuracy (Tsamardinos 2022; Eertink et al. 2022).

**Fig. 4:**
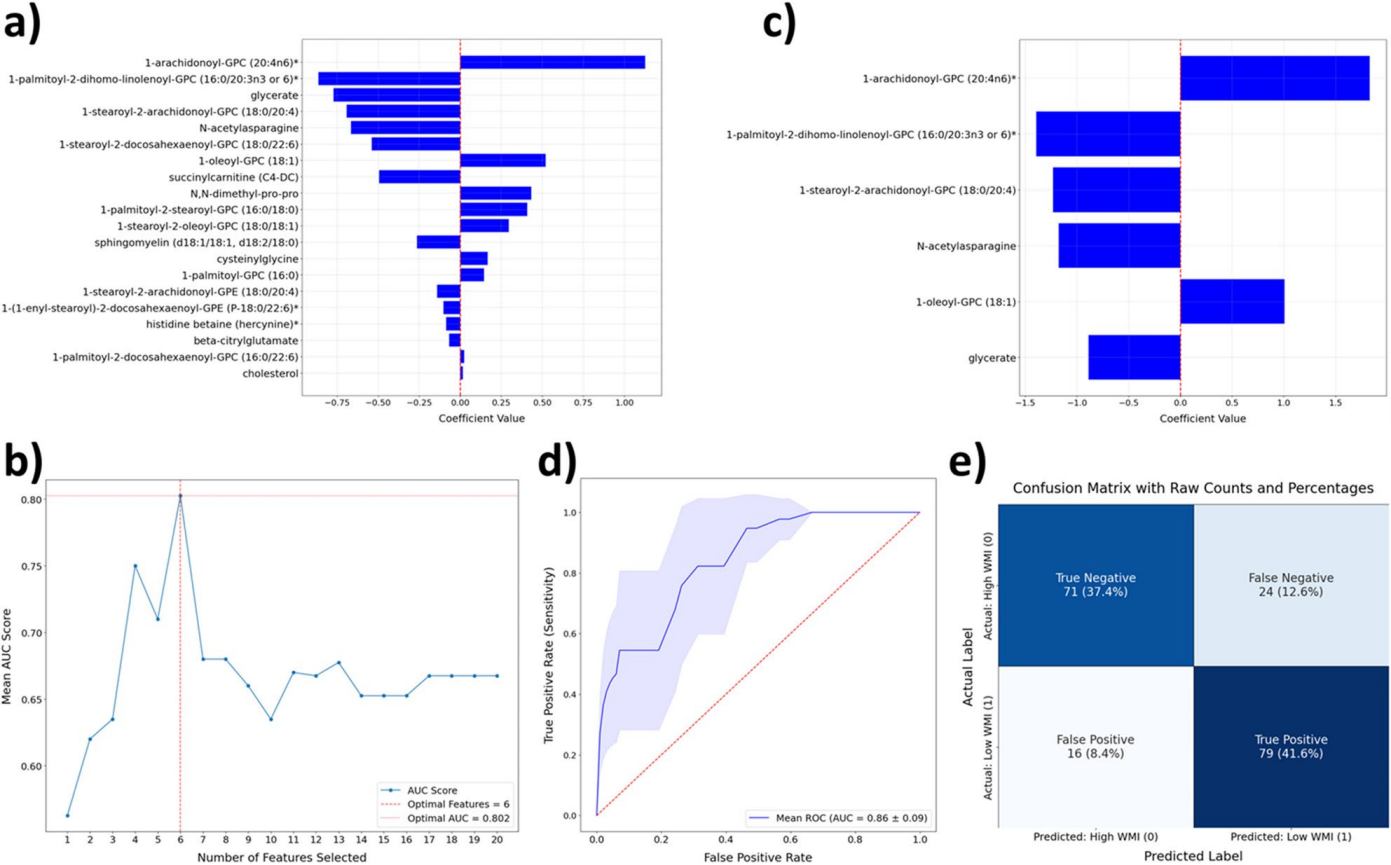
Metabolomic signature to predict low WMI at 12 weeks post-diagnosis. Machine learning was applied to predict a low WMI at T3 (12 weeks post-diagnosis) using a subset of 20 metabolites initially filtered by MEBA. The estimated β coefficients for these initial metabolites are shown in panel **a**). Recursive feature elimination with cross-validation (RFECV) was then used to determine **b**) the optimal number of metabolites for classification, using the area under the ROC curve (AUC) as the scoring metric. After feature selection and hyperparameter tuning, the estimated β coefficients for the final set of six metabolites are shown in panel **c**). The performance of the final model is illustrated by **d)** the average ROC curve and **e**) the confusion matrix, highlighting the classifier’s ability to discriminate between low-WMI patients and high-WMI patients

The T3 abundance of the top 20 metabolites ranked by Hotelling’s T^2^ statistic was used to train the model and compute β coefficients, indicating which metabolites were the most informative for distinguishing between the low- and high-WMI groups. Model performance was evaluated using the ROC-AUC as the scoring metric. This preliminary analysis indicated that several metabolites contributed minimally to the model, prompting further feature selection to improve its performance (Fig. 4-a). Therefore, we performed feature selection using recursive feature elimination (RFE) with cross-validation to reinforce model stability and retain only the metabolites contributing most to its predictive performance. Using this approach, we found that the optimal AUC was reached with a final set of six out of the 20 initial metabolites (Fig. 4-b): 1-arachidonoyl-GPC (20:4n6)* (estimated β: 1.83), 1-palmitoyl-2-dihomo-linolenoyl-GPC (16:0/20:3n3 or 6)* (estimated β: −1.39), 1-stearoyl-2-arachidonoyl-GPC (18:0/20:4) (estimated β: −1.23), n-acetylasparagine (estimated β: −1.17), 1-oleoyl-GPC (18:1) (estimated β: 1.01), and glycerate (estimated β: −0.89) (Fig. 4-c).

Following feature selection, the final list of six metabolites (Fig. 4-c) was used for hyperparameter tuning and threshold determination, using the AUC and F1 score and balancing precision and recall as performance metrics, respectively (Fig. 3). The final logistic regression model was then trained and evaluated using the optimized parameters, and its performance was assessed through the final ROC curve and confusion matrix (Fig. 4-d, e). Our final model achieved a high level of predictive performance after optimization (Fig. 4-c, AUC = 0.86 ± 0.09, *p* ≤ 0.001), indicating that in 86% of randomly selected patient pairs, one with a low WMI and one with a high WMI, the model correctly ranks the patient with low WMI above the patient with a high WMI. Interestingly, not all individual metabolites within this signature displayed significant differences in abundance between low- and high-WMI patients (Supp. Figure 7), highlighting that our model’s predictive power arises from the combined contribution of these metabolites rather than from isolated effects. In addition, as shown by the confusion matrix, our model exhibits a false positive rate of only 8.4% and a false negative rate of 12.6%, corresponding to an overall accuracy of 79%, further demonstrating its strong predictive ability (Fig. 4-e). Taken together, this metabolomic signature robustly predicts a low WMI in our cohort of 45 pediatric patients treated for ALL on DFCI 16–001.

WMI status can also be classified using classical inference-based logistic regression, which can estimate statistical associations between metabolites and WMI status but is not designed for predictive performance. To assess the stability and interpretability of our ML-based model, we compared it with the inference-based model using the same six metabolites selected by RFE (Supp. Figure 7-a, b). The β coefficients estimated from our ML logistic regression closely aligned with those obtained from the inference model, both in directionality and relative magnitude. Notably, several features that did not reach statistical significance in the inference model (1-palmitoyl-2-dihomo-linolenoyl-GPC (16:0/20:3n3 or 6)*, *p* = 0.074; 1-stearoyl-2-arachidonoyl-GPC, *p* = 0.108; glycerate, *p* = 0.171) remained key contributors to the predictive performance of the ML model. This comparison demonstrates that the predictive signature is driven by a stable and biologically interpretable pattern that persists across both machine learning and classical statistical modeling approaches.

## Discussion

The molecular mechanisms underlying cancer-related cognitive impairment remain poorly understood. Compounding this issue, late detection of cognitive deficits in survivors limits the effectiveness of potential interventions. In this study, we present the results of a longitudinal metabolomic analysis of CSF from 45 pediatric ALL patients enrolled on the DFCI 16–001. The results revealed global metabolomic shifts during the first 18 days of treatment. Additionally, alterations in major metabolic pathways were revealed, which, importantly, were sustained across five timepoints spanning the first 20 weeks of leukemia treatment. Lastly, using a logistic regression‑based ML model, we identified a lipid-rich metabolomic signature in CSF collected 12 weeks post‑diagnosis predictive of low WMI after therapy completion, thereby paving the way for the early detection of CRCI in pediatric ALL patients.

Our results based on dimensionality reduction and pathway analyses demonstrate a large-scale reorganization in the CSF metabolome during the first 18 days of treatment (T0 to T1). This underscores the metabolic burden of the chemotherapy induction phase, which precedes the remission typically reached by T2 (11 weeks post-diagnosis), consistent with prior finding from DFCI 11–001 showing that 97% of patients were in remission by day 32 (Lm et al. 2025). Several explanations may account for these changes. One possibility is that chemotherapy induction clears most leukemia cells by day 18, allowing the CNS to revert toward a non-pathological metabolic state. However, our UMAP findings do not support this hypothesis, as it indicates that the global profile of the CSF metabolome at T0 closely resembled that at T2–T4, suggesting that the metabolic state at T0 was not substantially impacted by leukemia.

An alternative explanation is that the metabolic shift occurring between T0 and T1 reflects tumor lysis syndrome (TLS), a well-recognized complication of early chemotherapy. TLS results from the rapid breakdown of tumor cells and can produce abrupt metabolic changes detectable in blood and CSF (Adeyinka et al. 2025). This mechanism would explain why comparable metabolic variations are not observed at later timepoints. Taken together, these findings highlight the importance of CSF as a matrix for investigating chemotherapy neurotoxicity. By appropriately reflecting brain homeostasis and the effects of treatment, CSF provides a powerful window into the metabolic alterations induced by chemotherapy (Gottfries et al. 2001; Dayon et al. 2017; Zhou et al. 2023).

The CSF of pediatric patients undergoing chemotherapy also exhibited temporal changes in lipid, amino acid, and one-carbon pathways, with phospholipid-related changes standing out as the most noteworthy. Specifically, phosphatidylcholines and phosphatidylethanolamines were affected by chemotherapy at all timepoints. These results are consistent with previous studies reporting pronounced alterations in phospholipid serum levels in childhood ALL survivors compared with healthy controls (Morel et al. 2017; Yan et al. 2025). Similarly, in long-term survivors of childhood ALL, increased CSF levels of sphingomyelin and lysophospholipids have been measured following induction and consolidation; or induction alone, respectively (Krull et al. 2013). Notably, our results also show that lipids are underrepresented among metabolites significantly altered between T0 and T2 and overrepresented between T2 and T4, demonstrating the direct consequences of chemotherapy on CSF lipid metabolism in pediatric ALL patients in remission.

Disruptions in one-carbon metabolism over the course of treatment were anticipated as the antifolate methotrexate, a key component of modern ALL chemotherapy regimens (Hunger and Mullighan 2015; Inaba et al. 2013; Inaba and Mullighan 2020), was administered to patients intrathecally on days 18 and 32 of induction, and again at later stages of therapy as detailed in Supp. Table 1. Pediatric ALL patients’ brains are still undergoing development and maturation. This window of susceptibility increases their vulnerability to the well-documented detrimental cellular and functional consequences of one-carbon metabolism disruptions (Willekens et al. 2019; Willekens et al. 2023; El Hajj et al. 2014). This expected result supports the internal consistency of our metabolomic findings and reiterates the need to develop approaches that can prevent or mitigate CRCI.

The second major aim of this study was to identify early biomarkers predictive of CRCI in a selected subset of 45 pediatric ALL patients enrolled on DFCI 16–001 and chosen based on high or low WMI one year after therapy completion. Given that CRCI has been consistently associated with working memory deficits (Williams et al. 2016; Stewart et al. 2006; Peterson et al. 2008), which are themselves linked with poorer academic performance (Maehler and Schuchardt 2016), WMI was selected as a clinically relevant indicator of a key cognitive domain affected by chemotherapy. Unlike the longitudinal changes observed across treatment timepoints, neither our PCA nor UMAP analysis revealed broad shifts in the CSF metabolome of pediatric ALL patients based on WMI status (Supp. Figure b). However, for the top 20 metabolites distinguishing low and high WMI over the day-18 to 20-week window, the strongest differences were observed at 12 weeks (T3). Using the T3 abundance of these metabolites as input features, our logistic regression–based ML model revealed a metabolomic signature composed of six metabolites (1-arachidonoyl-GPC (20:4n6)*, 1-palmitoyl-2-dihomo-linolenoyl-GPC (16:0/20:3n3 or 6)*, 1-stearoyl-2-arachidonoyl-GPC (18:0/20:4), n-acetylasparagine, 1-oleoyl-GPC (18:1), and glycerate) that accurately predicted low WMI in our cohort of patients.

Importantly, the composition of this signature reveals biological relevance. This is reflected by the presence of four glycerophosphocholines in this signature, which is a class of lysophospholipids previously linked to Alzheimer’s disease (AD) and multiple sclerosis (Law et al. 2019). Phospholipids are critical for membrane integrity and synaptic function, and their CSF levels are altered in various neurological disorders (Fonteh et al. 2013; Wood and Woltjer 2022; Xu et al. 2024). Consistently, CSF phosphatidylcholines have been associated with diverse neurological conditions, including CNS infection, ALS, traumatic brain injury, and Parkinson’s disease (Al-Mekhlafi et al. 2021; Blasco et al. 2017; Pasvogel et al. 2010; Qiu et al. 2023). Previous studies have also reported increased oxidation of glycerophosphocholines in the CSF of pediatric ALL patients, but this aspect could not be assessed with the analytical approach used here (Caron et al. 2009; Stenzel et al. 2010; Ki Moore et al. 2015). Taken together with the results from our pathway analysis, these findings underscore a potential role for glycerophosphocholines in chemotherapy-induced neurotoxicity and suggest that future lipidomic studies may clarify the mechanistic contribution of these lysophospholipids to the pathophysiology of CRCI.

There are nevertheless a few notable limitations to this work that will be addressed in a future confirmatory study. First, the overall sample size was limited across timepoints, with T0 being particularly constrained, which reduces the power to draw definitive conclusions about the reliability and generalizability of the findings. Limited sample availability also did not allow the generation of independent discovery and validation cohorts. As a result, all available samples were used for model development, with performance estimated through stratified repeated cross-validation. Future studies will require larger cohorts with available 12-week CSF samples to enable true external validation and to confirm the generalizability of the six-metabolite signature. Second, while WMI is a clinically relevant indicator of cognitive function, it captures only a subset of CRCI manifestations; future studies should investigate additional cognitive domains to provide a more comprehensive assessment of the effects of chemotherapy on the pediatric brain. Third, an inherent limitation to studies focusing on cancer treatment toxicity lies in the absence of a pretreatment baseline and the lack of an external control group, which constrains causal inferences regarding the specific contribution of chemotherapy to the observed cognitive outcomes.

The demographic and clinical characteristics of the two WMI groups were generally well balanced, except for an overrepresentation of “Hispanic or Latino” individuals in the low WMI group (Table 2). Because our ML model was stratified by WMI but not by ethnicity to preserve stability and avoid overfitting, this imbalance could bias the model towards population-specific features. Larger and more ethnically balanced cohorts will be necessary to confirm the generalizability of the metabolomic signature.

Despite these limitations, this study establishes a robust proof-of-concept: it delineates a set of predictive metabolites, identifies T3 as the most informative timepoint, and lays the foundation for a targeted, higher-throughput strategy. Future work will focus on expanding patient cohorts, validating the six-metabolite panel at T3, and incorporating lipidomics and oxidation-specific analyses.

Although the concept of the metabolome emerged at the end of the 1990 s (Oliver et al. 1998), metabolomics is still considered an emerging field by many. This is partly due to the cost of large-scale metabolomic profiling, the lack of standardized analytical pipelines across institutions, and the limited biological knowledge available for many metabolites. Thus, despite invaluable resources such as the Human Metabolome Database (Wishart et al. 2007), these challenges have constrained the development of large discovery and validation cohorts and have also limited our ability to map individual metabolites to specific biological processes with the same confidence achieved in transcriptomics or proteomics.

In the context of chemotherapy-related cognitive impairment, this challenge is amplified by the heterogeneity of clinical presentations, as CRCI can affect multiple cognitive domains. Patients may also exhibit diverse structural and functional changes in the brain (Spencer Noakes et al. 2018; Lövblad et al. 1998; Mergen et al. 2022; Elens et al. 2021), and risk factors are multifactorial, including individual polymorphisms (Cole et al. 2015; Bhojwani et al. 2014). Future predictive models will therefore require integrating CSF metabolomics with complementary modalities such as MRI-based measures, magnetic resonance spectroscopy (MRS) to capture regional metabolic changes within the brain (Hirokawa et al. 2025), and genetic risk factors, alongside longitudinal assessments of multiple cognitive domains. Integrating these modalities will enable a better understanding of CRCI risk and mechanisms.

Because of this complexity, developing such multimodal predictive models will require large patient cohorts and coordinated efforts across multiple institutions. Nonetheless, pediatric ALL survivors urgently need simpler approaches that, even if they do not capture the full complexity of CRCI, can identify children at elevated risk for cognitive difficulties following chemotherapy. Our findings contribute to this objective by demonstrating that early CSF metabolomic changes contain predictive information that motivates deeper investigation in expanded cohorts or through multimodal strategies.

## Conclusions

In summary, this study highlights dynamic metabolic changes during the first 20 weeks of chemotherapy in pediatric ALL patients and identifies a metabolomic signature with strong potential for the early identification of individuals at risk for CRCI, particularly regarding working memory deficits. While external validation is essential, the model’s predictive accuracy and biological relevance support its value as a candidate screening tool during a critical treatment window when intervention may be most effective. Beyond advancing our understanding of CRCI pathophysiology, these findings may also inform early diagnostic strategies and contribute to improving long-term cognitive outcomes and quality of life in pediatric cancer survivors.

## Supplementary Information

Supplementary Material 1.

## Data Availability

The datasets used and/or analyzed during the current study are available from the corresponding author on reasonable request.

## Abbreviations

AD: Alzheimer's disease
ALL: Acute lymphoblastic leukemia
AUC: Area under the curve
CNS: Central nervous system
CRCI: Chemotherapy-related cognitive impairment
CSF: Cerebrospinal fluid
DFCI: Dana-Farber Cancer Institute
EDA: Exploratory data analysis
FDR: False discovery rate
GPC: Glycerophosphocholine
IT: Intrathecal
KEGG: Kyoto Encyclopedia of Genes and Genomes
MEBA: Multivariate empirical Bayes analysis
ML: Machine learning
MRS: Magnetic resonance spectroscopy
PCA: Principal Component Analysis
RFE: Recursive feature elimination
RI: Retention time/index
ROC: Receiver operating curve
SD: Standard deviation
TLS: Tumor lysis syndrome
UMAP: Uniform manifold approximation and projection
UPLC-MS/MS: Ultrahigh-Performance liquid chromatography-tandem mass spectrometry
WBC: White blood cell count
WMI: Working memory index
