# Supplementary material for: CSF metabolomic signature during therapy for childhood acute lymphoblastic leukemia predicts subsequent working memory impairment
Source: Mol Med. 2025 Dec 30;32:12. doi: 10.1186/s10020-025-01414-z (PMC12865937; doi:10.1186/s10020-025-01414-z)
Supplement: Supplementary file 1 — Supplementary Material 1. [file 10020_2025_1414_MOESM1_ESM.docx]

# Supplementary Figures and Tables

| **Treatment Phase** | **Phase Duration** | **Day (relative to phase start)** | **Intrathecal Chemotherapy** | | | | | |
| --- | --- | --- | --- | --- | --- | --- | --- | --- |
| Pre-treatment |  | Day 0 |  | - IT cytarabine | | | | |
| Induction 1A | 32 days | Day 18 |  | - Triple IT | | | | |
|  |  | Day 32 |  | - IT methotrexate | | | | |
| Induction 1B | 3–6 weeks |  |  | - no IT chemotherapy | | | | |
| Consolidation 1A | 21–28 days | Day 1 |  | - IT methotrexate | | | | |
| Consolidation 1B | 7 weeks | Day 1 |  | Very High-Risk patients - IT methotrexate | | | | |
| Consolidation 1C |  | Day 1 |  | Very High-Risk patients - IT methotrexate | | | | |
| CNS phase | 21 days | Day 1 |  | - Triple IT | | | | |
|  |  |  |  | CNS3 patients - CNS radiation | | | | |
|  |  | Day 4 |  | - Triple IT | | | | |
|  |  | Day 8 |  | - Triple IT | | | | |
|  |  | Day 10 |  | CNS3 patients - CNS radiation | | | | |
|  |  | Day 11 |  | - Triple IT | | | | |
| Consolidation 2 | 30 weeks |  |  | - Every 9 weeks: Triple IT | | | | |
| Continuation | Until 52 weeks  of complete remission |  |  | B-lineage patients  - Every 9 weeks x 6 doses : Triple IT  - Every 18 weeks : Triple IT |  | T-lineage patients  - Every 9 weeks x 6 doses : Triple IT |  | CNS3 patients  (regardless of immunophenotype)  - Every 18 weeks: Triple IT |
|  |  |  |  |  |  |  |  |  |
|  |  |  |  |  |  |  |  |  |
| End of therapy |  |  |  |  |  |  |  |  |

**Supplementary Table 1: Intrathecal chemotherapy schedule**

IT: Intrathecal; Triple IT: Triple intrathecal chemotherapy (methotrexate, cytarabine, and hydroxycortisone)


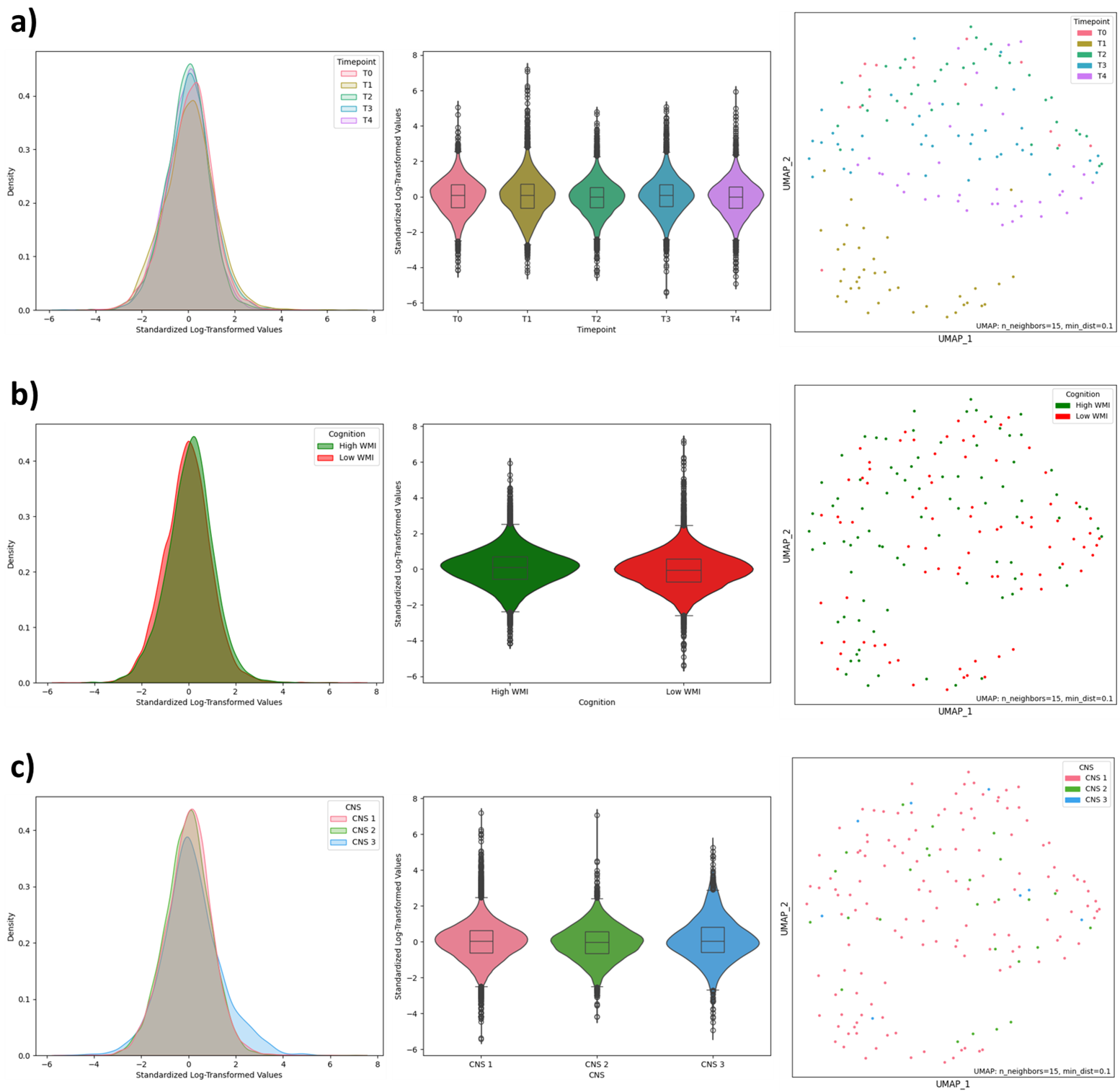


**Supplementary Figure 1: Exploratory data analysis by timepoint, WMI status, and CNS status.**

Exploratory data analysis stratified by **(a)** timepoint, **(b)** WMI status, and **(c)** CNS status. For each stratification, the left panels show kernel density plots of the standardized log-transformed abundance of 280 metabolites measured in the cerebrospinal fluid of 45 patients enrolled in DFCI 16–001. To facilitate comparison of distributional and variance patterns, the middle panels display the same data as violin plots. The right panels present the global data structure visualized using uniform manifold approximation and projection (UMAP) dimensionality reduction.


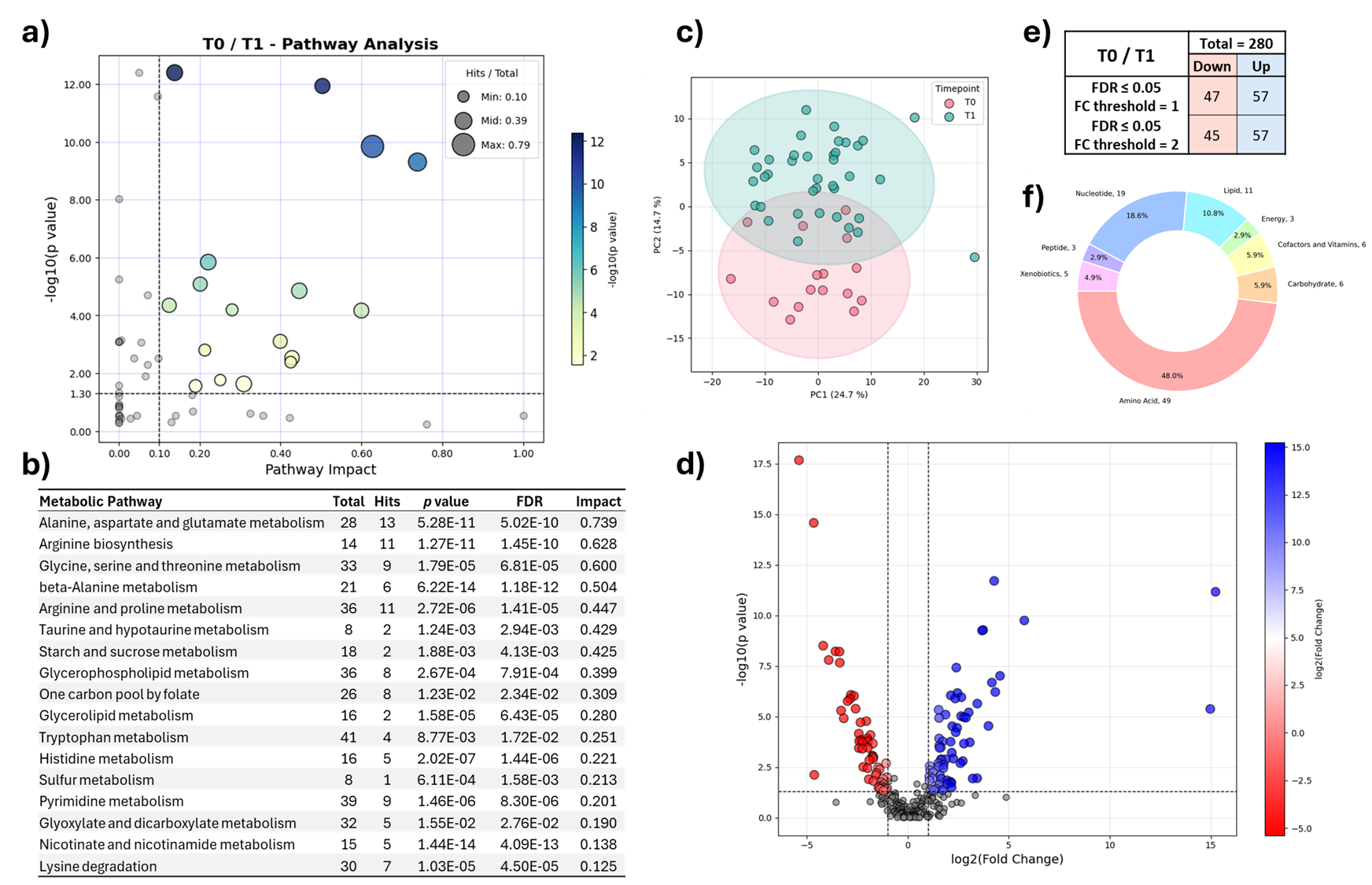


**Supplementary Figure 2: Metabolomic changes in the CSF of pediatric ALL patients between T0 and T1.**

Untargeted metabolomic profiling of CSF was performed on 45 pediatric patients diagnosed with ALL and treated on DFCI 16–001. A curated set of 280 metabolites was used to identify metabolic pathways enriched and impacted between T0 (day of diagnosis) and T1 (18 days post-diagnosis) through a combination of overrepresentation and pathway topology analyses. All comparisons are reported as T1 relative to T0; increases indicate higher levels at T1.

The pathway impact plot **(a)** highlights significantly enriched pathways (FDR ≤ 0.05) with a pathway impact score ≥ 0.10, with bubble size indicating the proportion of matched metabolites (hits) within each pathway. These retained pathways are summarized in panel **(b)**. Principal component analysis (PCA) **(c)**, including Hotelling’s T² confidence ellipses, was used to visualize the global distribution of samples at T0 and T1. Individual metabolites showing significant changes (FDR ≤ 0.05; fold change ≥ 2) are shown in the volcano plot **(d)** and summarized in panel **(e)**. The classification of these significant metabolites by biochemical class (superpathway) is illustrated in the donut chart **(f)**.


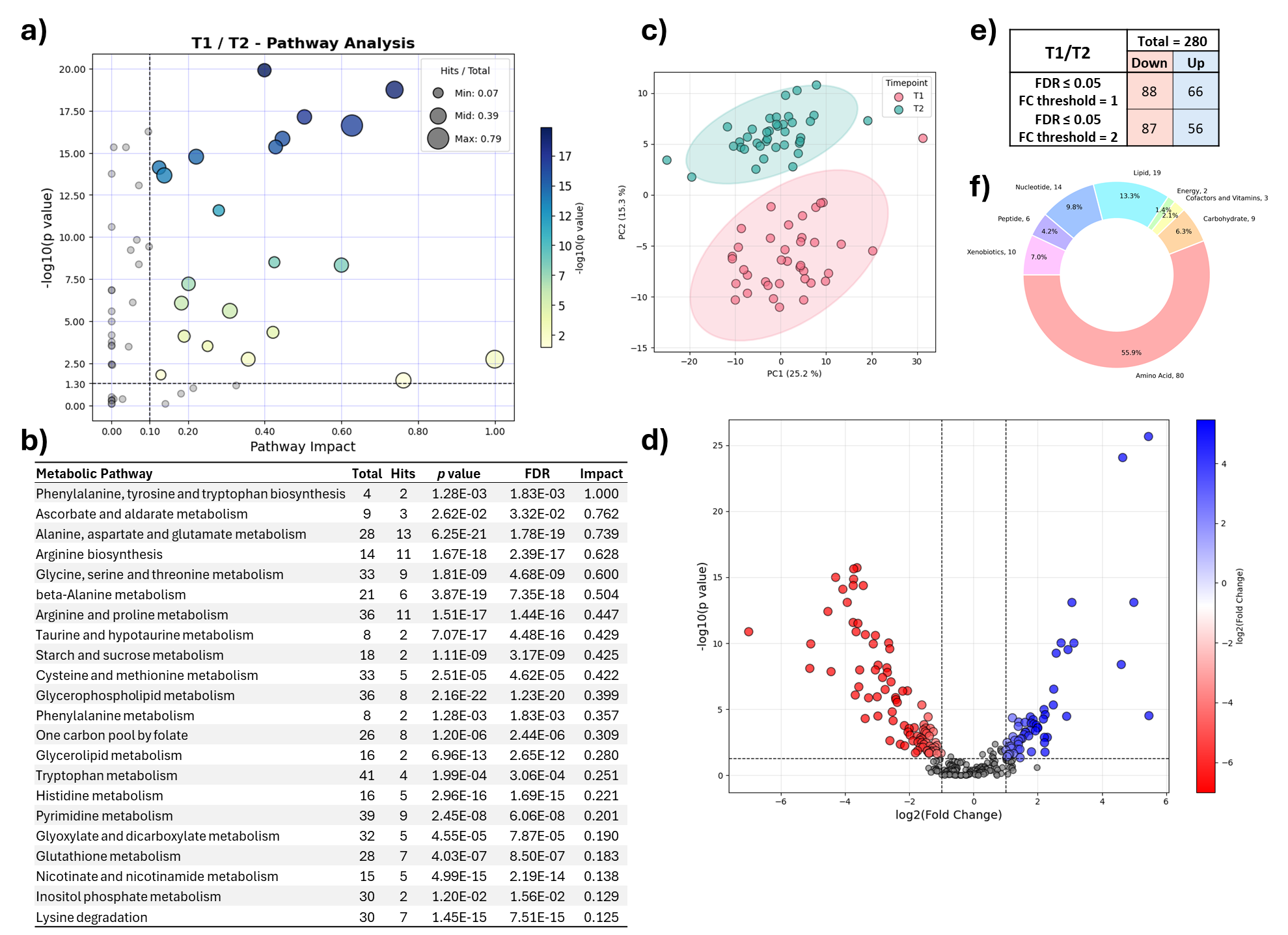


**Supplementary Figure 3: Metabolomic changes in the CSF of pediatric ALL patients between T1 and T2.**

Untargeted metabolomic profiling of CSF was performed on 45 pediatric patients diagnosed with ALL and treated on DFCI 16–001. A curated set of 280 metabolites was used to identify metabolic pathways enriched and impacted between T1 (18 days post-diagnosis) and T2 (11 weeks post-diagnosis) through a combination of overrepresentation and pathway topology analyses. All comparisons are reported as T2 relative to T1; increases indicate higher levels at T2.

The pathway impact plot **(a)** highlights significantly enriched pathways (FDR ≤ 0.05) with a pathway impact score ≥ 0.10, with bubble size indicating the proportion of matched metabolites (hits) within each pathway. These retained pathways are summarized in panel **(b)**. Principal component analysis (PCA) **(c)**, including Hotelling’s T² confidence ellipses, was used to visualize the global distribution of samples at T1 and T2. Individual metabolites showing significant changes (FDR ≤ 0.05; fold change ≥ 2) are shown in the volcano plot **(d)** and summarized in panel **(e)**. The classification of these significant metabolites by biochemical class (superpathway) is illustrated in the donut chart **(f)**.


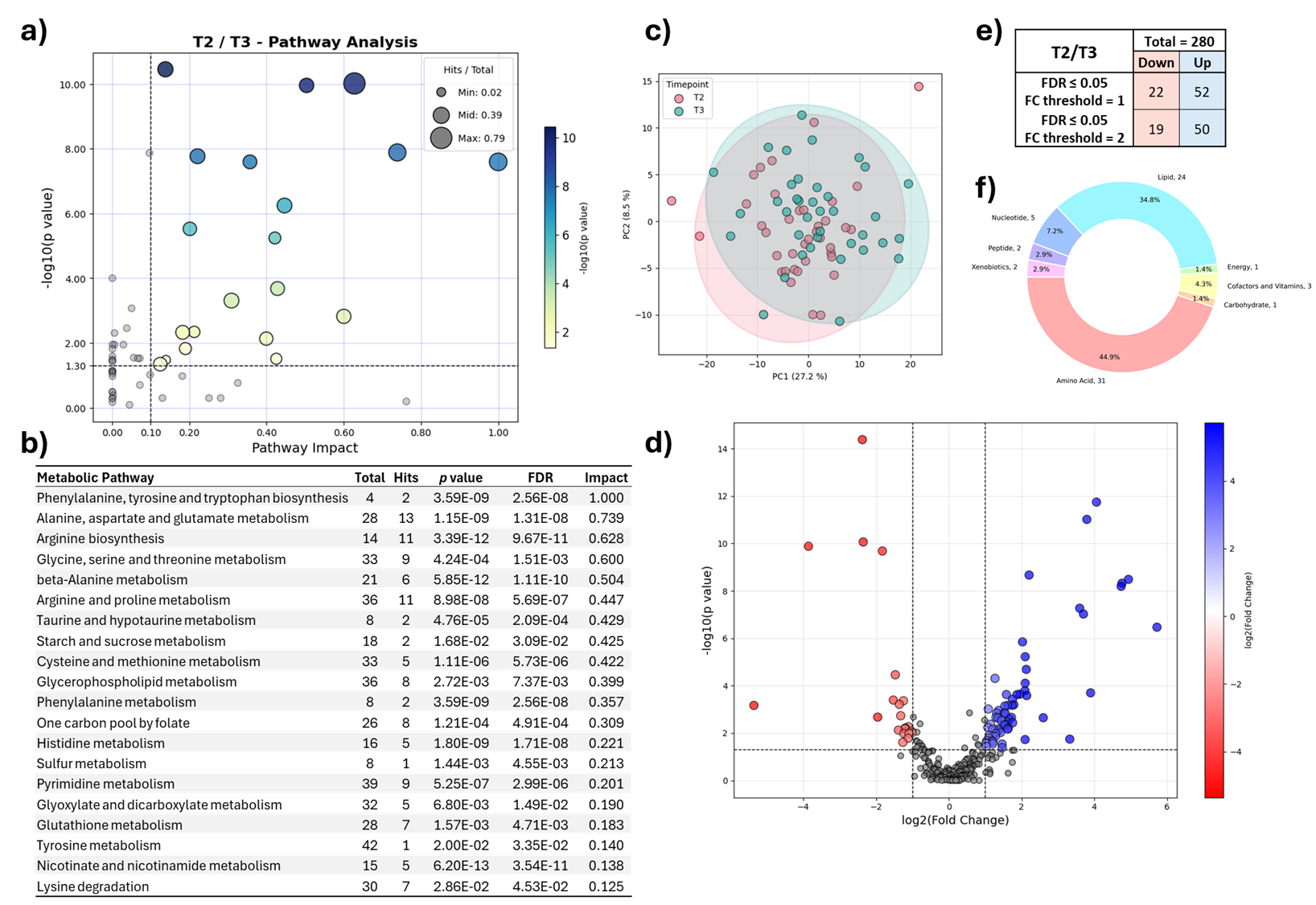


**Supplementary Figure 4: Metabolomic changes in the CSF of pediatric ALL patients between T2 and T3.**

Untargeted metabolomic profiling of CSF was performed on 45 pediatric patients diagnosed with ALL and treated on DFCI 16–001. A curated set of 280 metabolites was used to identify metabolic pathways enriched and impacted between T2 (11 weeks post-diagnosis) and T3 (12 weeks post-diagnosis) through a combination of overrepresentation and pathway topology analyses. All comparisons are reported as T3 relative to T2; increases indicate higher levels at T3.

The pathway impact plot **(a)** highlights significantly enriched pathways (FDR ≤ 0.05) with a pathway impact score ≥ 0.10, with bubble size indicating the proportion of matched metabolites (hits) within each pathway. These retained pathways are summarized in panel **(b)**. Principal component analysis (PCA) **(c)**, including Hotelling’s T² confidence ellipses, was used to visualize the global distribution of samples at T2 and T3. Individual metabolites showing significant changes (FDR ≤ 0.05; fold change ≥ 2) are shown in the volcano plot **(d)** and summarized in panel **(e)**. The classification of these significant metabolites by biochemical class (superpathway) is illustrated in the donut chart **(f)**.


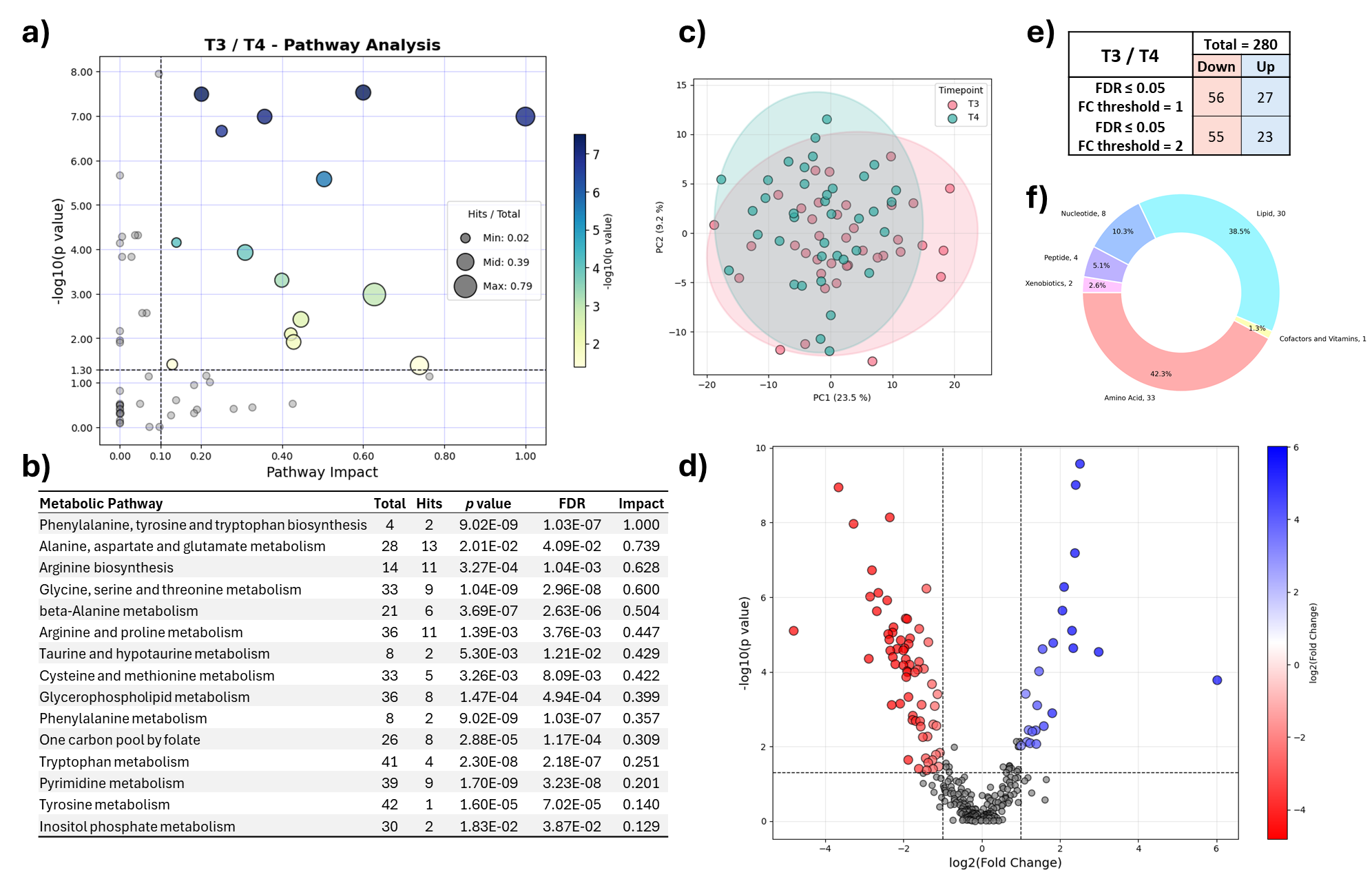


**Supplementary Figure 5: Metabolomic changes in the CSF of pediatric ALL patients between T3 and T4.**

Untargeted metabolomic profiling of CSF was performed on 45 pediatric patients diagnosed with ALL and treated on DFCI 16–001. A curated set of 280 metabolites was used to identify metabolic pathways enriched and impacted between T3 (12 weeks post-diagnosis) and T4 (20 weeks post-diagnosis) through a combination of overrepresentation and pathway topology analyses. All comparisons are reported as T4 relative to T3; increases indicate higher levels at T4.

The pathway impact plot **(a)** highlights significantly enriched pathways (FDR ≤ 0.05) with a pathway impact score ≥ 0.10, with bubble size indicating the proportion of matched metabolites (hits) within each pathway. These retained pathways are summarized in panel **(b)**. Principal component analysis (PCA) **(c)**, including Hotelling’s T² confidence ellipses, was used to visualize the global distribution of samples at T3 and T4. Individual metabolites showing significant changes (FDR ≤ 0.05; fold change ≥ 2) are shown in the volcano plot **(d)** and summarized in panel **(e)**. The classification of these significant metabolites by biochemical class (superpathway) is illustrated in the donut chart **(f)**.


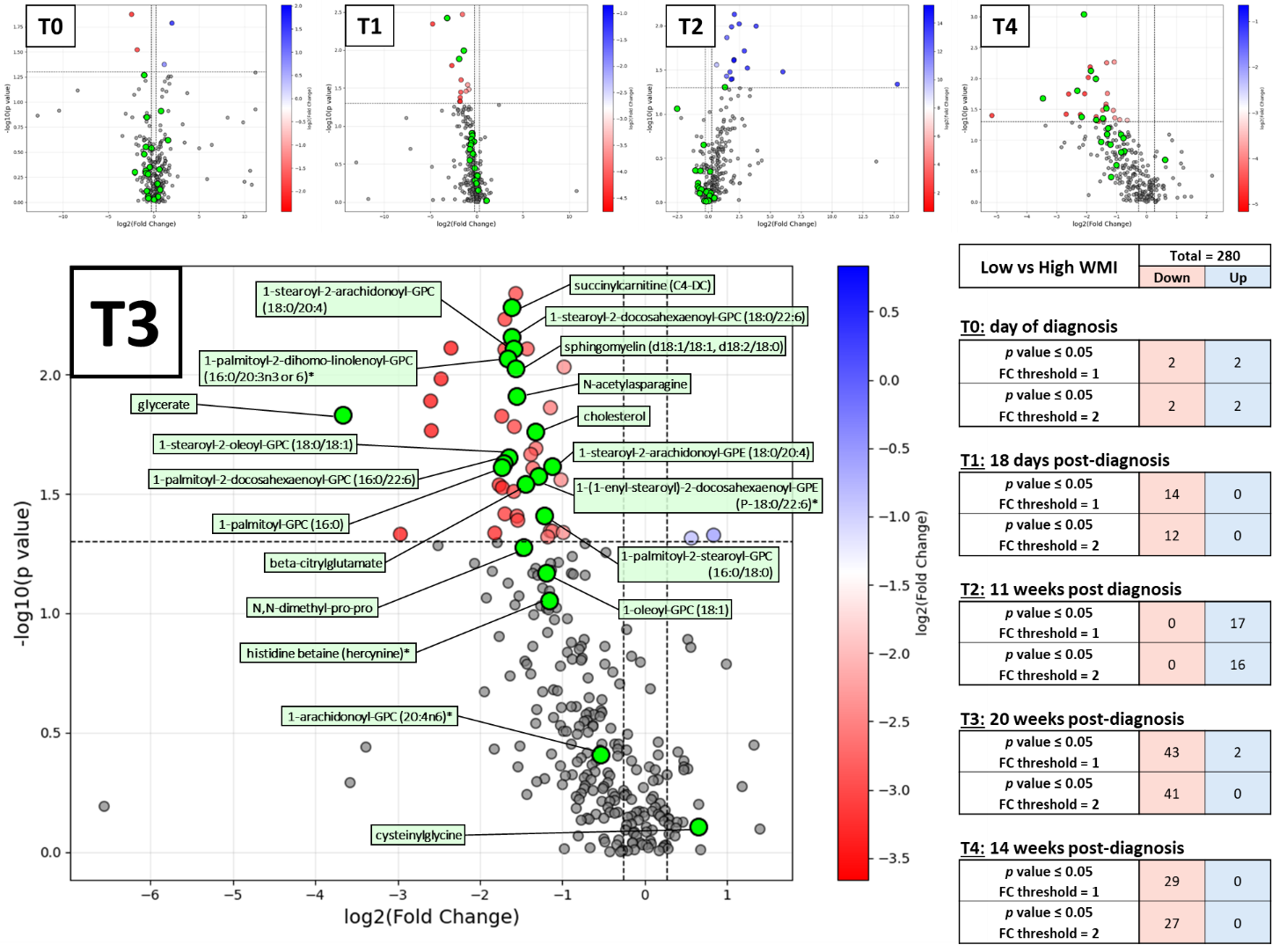


**Supplementary Figure 6: Longitudinal changes in the CSF metabolome of pediatric ALL patients stratified by WMI status.**

Untargeted metabolomic profiling of CSF was performed on 45 pediatric patients diagnosed with ALL and treated on DFCI 16–001. A curated set of 280 metabolites was used to assess differences in individual metabolite abundance between patients with high or low WMI, as assessed 1-2 years after the end of therapy. Volcano plots were used to visualize these differences at each timepoint, with significance defined by a *p* value ≤ 0.05 and a fold change ≥ 1. All comparisons are reported as Low WMI relative to High WMI; increases indicate higher levels in Low WMI. The timepoints are as follows: T0, day of diagnosis and first day of treatment; T1, 18 days post-diagnosis; T2, 11 weeks post-diagnosis; T3, 12 weeks post-diagnosis; and T4, 20 weeks post-diagnosis. The 20 metabolites identified as having the most distinct longitudinal profiles between WMI groups, on the basis of multivariate empirical Bayes analysis (MEBA), are highlighted in green. The T3 timepoint contained the greatest proportion of significant MEBA-filtered metabolites. Accordingly, the T3 volcano plot is displayed at a larger scale within the figure.

Summary tables to the right display the number of significantly different metabolites at each timepoint using two fold-change thresholds (≥1 and ≥2), with a consistent *p* value threshold of ≤ 0.05.


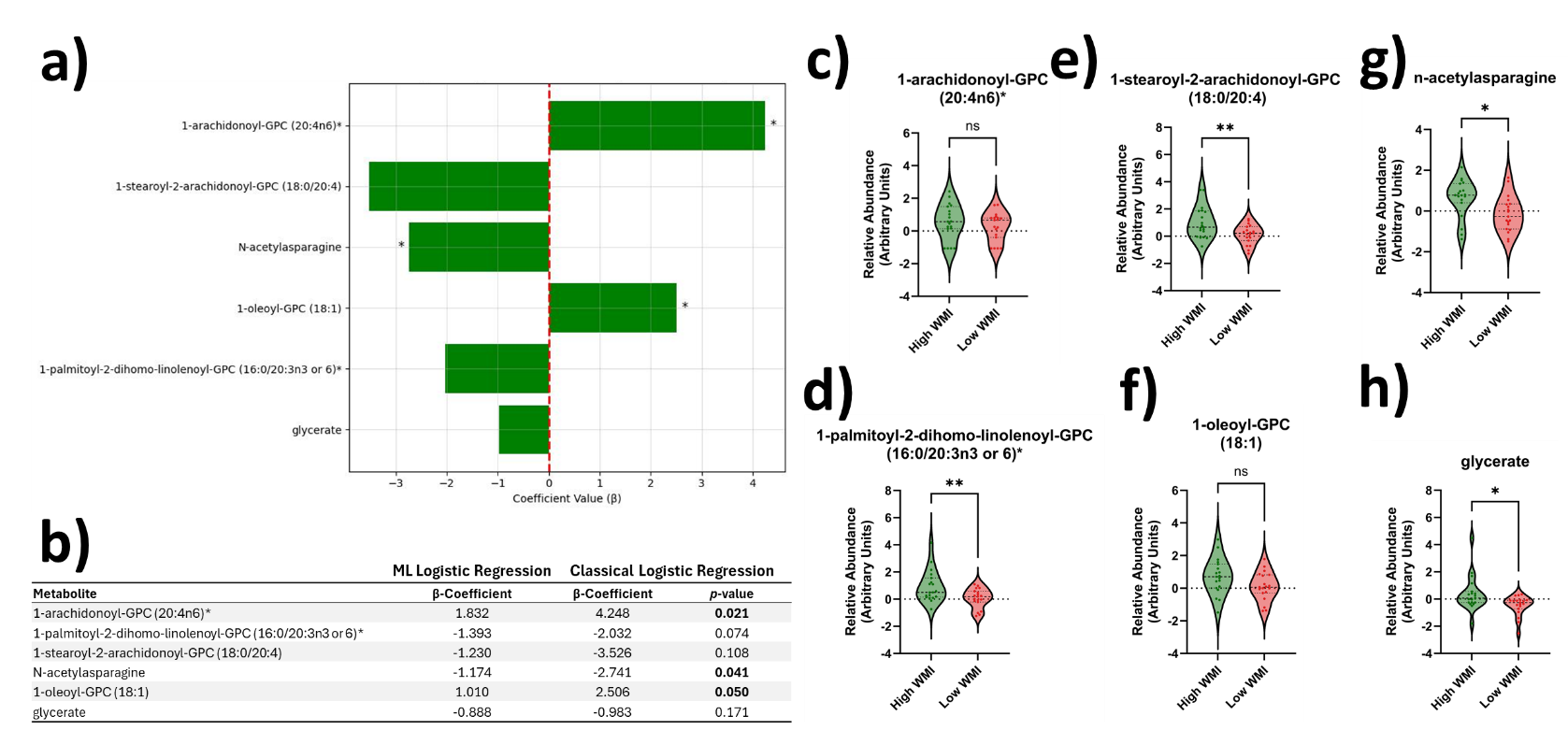


**Supplementary Figure 7: Comparison of ML-based logistic regression and classical inference-based logistic regression models.**

**a)** The β coefficients estimated by the ML-based logistic regression model were compared with those obtained via classical inference-based logistic regression. *P* values for the classical model were calculated using the Wald test (**p* ≤ 0.05). Panel **b)** illustrates the direct comparison of the β coefficients between the ML and classical models. The coefficient direction and relative magnitude are consistent across both models. Significant associations (*p* ≤ 0.05) are bolded. **c-h)** Violin plots of the relative abundances of the six selected metabolites in the CSF of high- and low-WMI patients. Statistical significance was assessed using Student’s t-test (**p* ≤ 0.05, ***p* ≤ 0.01).
